# Diets, Dietary Patterns, Single Foods and Pancreatic Cancer Risk: An Umbrella Review of Meta-Analyses

**DOI:** 10.3390/ijerph192214787

**Published:** 2022-11-10

**Authors:** Vincenza Gianfredi, Pietro Ferrara, Monica Dinu, Mariateresa Nardi, Daniele Nucci

**Affiliations:** 1 Department of Biomedical Sciences for Health, University of Milan, Via Pascal, 36, 20133 Milan, Italy; 2 CAPHRI Care and Public Health Research Institute, Maastricht University, 6200 MD Maastricht, The Netherlands; 3 Center for Public Health Research, University of Milan-Bicocca, 20900 Monza, Italy; 4 IRCCS Istituto Auxologico Italiano, 20145 Milan, Italy; 5 Department of Experimental and Clinical Medicine, University of Florence, 50134 Florence, Italy; 6 Nutritional Support Unit, Veneto Institute of Oncology IOV-IRCCS, Via Gattamelata, 64, 35128 Padua, Italy

**Keywords:** review, meta-analysis, umbrella review, pancreatic cancer, diet, dietary patterns, food

## Abstract

Pancreatic cancer (PC) represents the third leading cause of cancer death in 2020. Despite the fact that, in 2018, the World Cancer Research Fund report concluded that there is still a lack of evidence on the role of foods or diets and risk for PC, a flourishing body of evidence has been published and needs to be analyzed. For this reason, we conducted an umbrella review on the association between different dietary patterns/food components and PC. Data sources PubMed/MEDLINE, Scopus, Web of Science, EMBASE, and the Cochrane Collaboration were searched. The Joanna Briggs Institute Umbrella Review Methodology was used. The protocol was registered in PROSPERO. A total of 23 articles were included, covering a wide range of dietary patterns/food components: healthy/prudent dietary patterns (*n* = 4), Mediterranean diets (MedDiet) (*n =* 1), plant-based diets (*n =* 2), the Dietary Inflammatory Index (DII) (*n =* 2), western diets (*n =* 2), and, lastly, unhealthy diets (*n =* 2). Regarding dietary components, the following were assessed: total fruit (*n =* 2), citrus fruit (*n =* 1), total vegetables (*n =* 2), cruciferous vegetables (*n =* 1), red meat (*n =* 6), processed meat (*n =* 4), poultry (*n =* 2), eggs (*n =* 1), fish (*n* = 5), whole grain (*n =* 2), potato (*n =* 1), and nuts (*n =* 2). The methodological quality of the included meta-analyses was generally low or critically low. Although the strength of evidence was generally weak, convincing or suggestive evidence was found for a healthy/prudent, plant-based diet, fruit and vegetables, and lower risk of PC, whereas a high intake of red meat was associated with a higher risk of PC at a convincing level of evidence. Further studies are needed to confirm the role of the other dietary patterns/food components and the risk of PC.

## 1. Introduction

Pancreatic cancer (PC) is the 12th most common cancer type in the world, with 495.773 new cases in 2020. Although the incidence rate is not very high, PC is almost always intractable, and it represents the third leading cause of cancer death in high-income countries (213,863 deaths) and the eighth worldwide with 466.003 deaths (4.7% of all deaths caused by cancer) in 2020 [1]. Furthermore, the number of new cases of PC globally is foreseen to increase dramatically by about 70% in 2040, with a significant public health burden.

The high mortality rate of PC depends on: (i) difficulty in early diagnosis; (ii) absence or poor specificity of symptoms; (iii) pronounced ability of metastatic dissemination.

Although early diagnosis is crucial to increase the survival rate, no screening programs have been established for the asymptomatic population due to the low incidence and the lack of efficient methodology [2]. 

In this context, an overall understanding of the risk factors associated with PC becomes of primary importance to reduce incidence by the implementation of effective preventive strategies also focused on lifestyle. Indeed, along with genetic factors (heritability account for 21% of pancreatic cancers) [3] diabetes (those receiving a new diagnosis of diabetes have an 8-fold higher risk of developing pancreatic cancer) [4,5], and chronic pancreatitis (the risk of pancreatic cancer is up to 16 fold higher in within the first 2 years from the diagnosis of chronic pancreatitis compared those without) [6], PC risk is also associated with, other risk factors related to lifestyle. As reported by the World Cancer Research Fund and American Institute for Cancer Research in their last report dating back to 2018, smoking habits (25% of pancreatic cancers are caused by tobacco smoking) [7] and obesity (obese subjects have 20–50% increased risk compared to normal weight) [8,9], have been identified as a convincing cause of PC [5]. Moreover, consumption of other food components, such as red and processed meat, alcoholic drinks, foods and beverages containing fructose, and foods containing saturated fatty acids, have shown limited suggestive evidence in increasing the risk of PC.

What emerges from the latest update of the WCRF/AICR report is that there is still a lack of sufficient evidence on the possible role of many foods or dietary patterns as risk or protective factors for PC.

However, in recent years several studies have been published, and new evidence has emerged on the possible role of diet and its components in the prevention of PC [10,11,12,13]. There is, therefore, a need to analyze this important body of evidence. From this perspective, we conducted an umbrella review aimed at focusing on and summarizing all the available evidence from existing meta-analyses of both observational and intervention studies on the effects of different types of dietary patterns and food components on PC risk. This approach will provide an overview of the quality and validity of the associations studied while also assessing the strengths and limitations of the evidence.

## 2. Materials and Methods

The current umbrella review was registered in the PROSPERO International Prospective Register of Systematic Reviews database (ID number: CRD42022343451, at www.crd.york.ac.uk/PROSPERO, (accessed on 19 July 2022)). We planned, conducted, and reported this umbrella review of observational studies and randomized clinical trials in concordance with the methods recommended by the Cochrane Collaboration [14] (the Preferred Reporting Items for Systematic Reviews and Meta-Analyses PRISMA guidelines) [15] and documented the process and results in accordance with the methodology developed by The Joanna Briggs Institute Umbrella Review Methodology [16]. It was designed to answer the following question: “What is the strength and validity of the existing evidence assessing the association between dietary patterns (from both observational research and RCTs) and/or single food components (e.g., fish or fruit and vegetables, etc.) and PC risk among adults?”.

### 2.1. Search Strategy and Data Sources

Five electronic databases (PubMed/MEDLINE, Scopus, Web of Science, EMBASE, and the Cochrane Collaboration) were searched independently by two authors (VG and DN). A systematic search was conducted in order to retrieve and collect all the potential indexed articles published up to 20 July 2022. The search strategy was developed using a combination of Mesh and free-text words. The identified keywords were combined using Boolean operators AND and OR. The search strategy was first developed in PubMed and then adapted to other databases. No time filter was used; moreover, no restriction was adopted. The full search strategy for each database is set out in Supplementary Table S1. In addition, authors reviewed the references cited in the full-text articles, and in addition, experts in the field were consulted in order to retrieve any additional interesting articles. 

### 2.2. Inclusion/Exclusion Criteria

Two independent authors (VG, DN) reviewed titles and abstracts of all obtained articles and selected systematic reviews with meta-analysis that (i) assessed the association between dietary patterns or food components and PC risk (ii) in adults aged 18 years or over, (iii) reported summary effect size estimated such as odds ratios (ORs), hazard ratios (HRs), or risk ratios (RRs) and their corresponding 95% confident intervals (CIs). Moreover, only articles published in English were eligible. Regarding both exposure and outcome, all methods used to assess dietary intake or PC diagnosis were considered eligible. Study population was limited to adult age groups according to pancreatic cancer epidemiology in order to avoid underestimation of the true association between the risk of the disease and the exposure of interest [17].

On the contrary, different types of study (such as systematic reviews without meta-analysis or pooled analysis), meta-analyses not published as peer-reviewed meta-analyses in international scientific journals (such as book, book chapter, thesis), no full-text papers (abstract, conference paper, letter, commentary, note) or meta-analysis not reporting comprehensive data (e.g., effect size and 95% confidence intervals) were excluded. Moreover, since beverages or micro-macronutrients are not considered food components because the first is classified as drinks (and not foods) and the second is food compounds; studies assessing the association between those and PC were also excluded from this analysis. Inclusion/exclusion criteria were developed according to the PICOS (Participants, Interventions, Control, Outcomes, Study Design) principle, and details are reported in Table 1. Relevant articles were obtained in full and assessed against the inclusion/exclusion criteria. If an article presented meta-analyses for more than one dietary pattern/dietary intervention, each of these was included separately.

### 2.3. Selection Process and Data Extraction

After calibration exercises, teams of 2 reviewers (VG and DN) independently performed a two-step article selection process. The first step was based on the screening of titles and abstracts, aiming to identify potentially eligible studies. During the second step, the full texts of potentially eligible studies were retrieved and independently assessed for eligibility by two review team members (VG and DN). 

For each meta-analysis, the following relevant details were extracted: first author and year of publication, number and type of included studies, comparison, type of reported effect size (that is, relative risk/hazard ratio, odds ratio, mean difference), study population, type of quality tool used to assess risk of bias, number of events and total sample size, and maximally adjusted effect size measurements along with the corresponding 95% CI. 

Data extraction from each identified meta-analysis was conducted independently by two authors (VG and DN). A standard data extraction spreadsheet elaborated in Microsoft Excel^®^ for Windows (Redmond, WA, USA, 2007) was used to perform data extraction. The spreadsheet was pre-piloted on 5 randomly selected papers to ensure methodological concordance and used to systematically record qualitative and quantitative data extracted from the included meta-analyses. Data were grouped according to the type of dietary patterns and single food components assessed in observational studies. 

In case of any disagreements between investigators (VG and DN) during both selection process and data extraction, they were solved through discussion; if disagreement persisted, a third investigator (MD) was sought. 

### 2.4. Quality Assessment 

The methodological quality of the included meta-analyses was independently assessed by two authors (PF and DN). In case of disagreement, a third investigator (VG) was involved. The quality of the meta-analyses was evaluated by means of the “A MeaSurement Tool to Assess systematic Reviews 2” (AMSTAR-2) questionnaire. The AMSTAR-2 tool has 16 items in total, with an overall rating based on weaknesses in critical domains. Critical domains were as follows: definition of the PICO components (item 1), adequacy of the literature search (items 4), risk of bias from individual studies being included in the review (item 9), appropriateness of meta-analytical methods (item 11), consideration of risk of bias when presenting the results of the review (item 12), and assessment of presence of publication bias (item 15) [18]. The overall confidence rate ranged between high (in the event of no or only one non-critical weakness), moderate (in the event of more than one non-critical weakness), low (in the event of one critical weakness) and critically low (in the event of more than one critical weakness) [18]. 

### 2.5. Strengths of Evidence Assessment and Data Analysis

All the statistical analyses were conducted using the ProMeta3^®^ (Internovi srl, Cesenatico, Italy) software. For each included meta-analysis, the following data were calculated. Using both fixed-effects and random-effects models, we calculated the summary effect sizes and their confidence intervals (CIs) by 95%. Moreover, the 95% prediction interval (PI) was estimated for the summary random effects. The 95% PI further accounts for the degree of between-study heterogeneity and provides a range whereby there is a 95% confidence that the effect in a new study examining the same association lies within it. Statistical heterogeneity between studies was evaluated using the I^2^ test [19]. Values of I^2^ below 50% are considered not large, values between 50–75% are classified as substantial, and values above 75% are classified as considerable [20]. The Egger’s regression asymmetry test and the standard error (SE) of the effect size (under random effects) for the largest study of each meta-analysis were conducted in order to evaluate the small-study effects [21]. The largest study was defined on the basis of the smallest SE. Small-study effects were identified if both the *p*-value for Egger’s test being <0.10 and the largest study having smaller effect size compared to the summary effect size were fulfilled [22]. 

All the above were used to define the strengths of evidence by using the following criteria: significance at *p* ≤ 0.05 and *p* ≤ 0.001; inclusion of >500 or >1000 cases for binary outcomes (>2500 or >5000 total participants if the metric was continuous); absence of considerable heterogeneity (I^2^ < 75%); 95% PI excluding the null value and absence of small-study effects. A total of five categories were identified: (i) *Convincing evidence*: significance threshold reached at *p* ≤ 0.001 for both random- and fixed-effects calculation; >1000 cases (or >5000 total participants if the metric was continuous); no large heterogeneity between studies (I^2^ < 50%); 95% PI excluding the null value; no evidence of small-study effects. (ii) *Highly suggestive evidence*: significance threshold reached at *p* ≤ 0.001 for both random- and fixed-effects calculation; >1000 cases (or >5000 total participants if the metric was continuous); no considerable heterogeneity between studies (I^2^ = 50–75%). (iii) *Suggestive evidence*: significance threshold reached at *p* ≤ 0.001 for random-effect calculation; 500–1000 cases (or 2500–5000 total participants if the metric was continuous). (iv) *Weak evidence*: significance threshold reached at *p* ≤ 0.05 for random-effects calculation. (v) *No-evidence*: significance threshold not reached (*p* > 0.05). 

## 3. Results

### 3.1. Search Results

**Figure 1:**
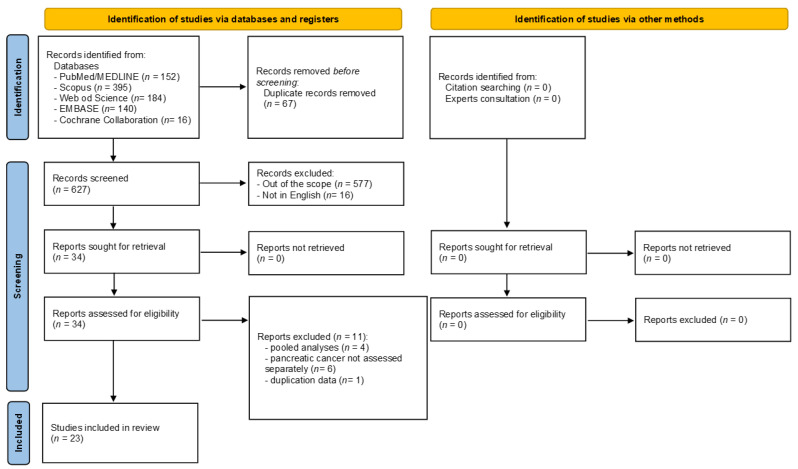
Flow diagram of the selection process.

Figure 1 shows the flow diagram, reporting the literature search process. The electronic search identified 887 articles in total, of which 152 were in PubMed/MEDLINE, 395 in Scopus, 184 in Web of Science, 140 in EMBASE, and 16 in the Cochrane Collaboration database. The selection process led to the immediate removal of 260 articles because of duplicates, leaving 627 articles. After reviewing the article titles, 577 were excluded for falling outside the scope of the current umbrella review, while 16 articles were removed, as they were not published in English. The 34 remaining articles were assessed in full-text, and 11 were excluded for the following reasons: where pooled analyses derived from consortium study and not from a systematic review (*n =* 4) [23,24,25,26,27]; PC risk not assessed separately (*n =* 6) [28,29,30,31,32,33], duplication data (*n =* 1) [34]. The reasons for exclusion are shown in Supplementary Table S2. At the end of the screening process, 23 articles met the inclusion criteria and were included in the current review [35,36,37,38,39,40,41,42,43,44,45,46,47,48,49,50,51,52,53,54,55,56,57]. All the included systematic reviews and meta-analyses only included original observational studies (case-control and/or cohort studies). Indeed, despite their inclusion/exclusion criteria (reported in Supplementary Table S3), at the end of the screening process, none of the retrieved meta-analyses included randomized clinical trials.

### 3.2. Characteristics and Methodological Quality of the Meta-Analyses Included

Table 2 reports detailed characteristics and methodological quality of the included meta-analyses. All the included articles were meta-analyses of observational studies (case-controls or cohort studies). Among the 23 articles included, 7 were studies assessing the association between dietary patterns and PC risk [35,39,40,43,48,52,56], whereas the remaining 16 studies analyzed the association between one or more dietary components and the risk of PC [36,37,38,41,42,44,45,46,47,49,50,51,53,54,55,57]. However, out of the 23 included articles, a total of 13 unique meta-analyses on dietary patterns and 29 unique meta-analyses on dietary components were detected because the articles reported the summary effect size separately by sex or study design. The oldest article retrieved was published in 1998 and assessed the association between whole grain and PC risk [42]. The two newest meta-analyses were published in 2022: one assessed two dietary components (poultry and fish) [38], and the other one assessed the association between plant-based diets and the risk of PC [56]. In all meta-analyses except one [41], authors compared the highest vs lowest dietary pattern adherence or diet component intake. The summary effect sizes (ES) pooled were Odds Ratio (OR) or Risk Ratio (RR) in most of the cases, apart from two meta-analyses where the authors estimated the Hazard Ratio (HR) [49,51]. Only in one meta-analysis did the author not specify the type of ES calculated [37]. Moreover, in five meta-analyses, the authors did not provide adequate data to estimate the summary ES using both fixed and random effects; for this reason, it was not possible to calculate the PI [37,41,42,43,46]. For these five meta-analyses, the ES reported in Table 2 are the only random-effects summary effect size as presented by the authors of the original meta-analysis.

All the meta-analyses were conducted on adults, including both men and women, except one study [57], where the association between red meat intake, processed meat intake, and PC risk was calculated separately for the two sexes. At the same, two studies [39,56] assessed the association between adherence to dietary patterns and PC risk based on study design. 

Regarding the dietary patterns, the following were analyzed: healthy/prudent dietary patterns (*n =* 4), the Mediterranean diet (MedDiet) (*n =* 1), a plant-based diet (*n =* 2), the Dietary Inflammatory Index (DII) (*n =* 2), a western diet (*n =* 2), and, lastly, unhealthy diet (*n =* 2). A detailed description of the diets/dietary patterns definitions adopted in each meta-analysis is reported in Supplementary Table S4. As for dietary components, the following were assessed: total fruit (*n =* 2), citrus fruit (*n =* 1), total vegetables (*n =* 2), cruciferous vegetables (*n =* 1), red meat (*n =* 6), processed meat (*n =* 4), poultry (*n =* 2), eggs (*n =* 1), fish (*n* = 5), whole grain (*n =* 2), potato (*n =* 1) and nuts (*n =* 2). The total numbers of dietary patterns and dietary components assessed are higher compared to the total number of included articles because some of them assessed more than one dietary pattern or dietary compound or because, in the same article, the authors performed more than one meta-analysis based on the study design or sex. 

A marked variability was observed in the included studies in the definitions of specific dietary patterns. For instance, both healthy, western, and unhealthy dietary patterns were defined based on a priori or a posteriori factor, on a cluster or principal components analysis, and in the meta-analysis performed by Grosso et al., different types of dietary patterns were combined all together [39]. For instance, a healthy diet, vegetarian dietary pattern, MedDiet pattern, and unsaturated fat dietary pattern were combined within the same meta-analysis. The same adherence to the MedDiet was quantified by means of different MedDiet scales [52]. On the contrary, very low or no heterogeneity was found when DII was considered since, in both cases, a univocal diet scale was in place. Regarding PC diagnosis, it was generally derived from electronic health records or histopathological diagnosis. 

The largest population (>1,000,000 subjects) was observed in meta-analyses on dietary components, such as total fruit [53] and citrus fruit [36], total vegetables [50] and cruciferous vegetable [47], red and processed meat [45,50,57], poultry [38] and fish [38,44,54]. On the other side, the smallest population was observed in a meta-analysis assessing the association between DII and PC risk (*n =* 2408 subjects) [43]. Only two of the meta-analyses did not assess the quality of the original studies included [42,45]. However, all the remaining studies but one used a validated tool [51]. Indeed, Qin 2012 assessed the risk of bias by setting four criteria defined a priori by the authors. The methodological quality of the meta-analyses included, determined by the AMSTAR-2 questionnaire, was moderate in 5 articles, low in 10, and critically low in 7 (Table 2). The main criticisms were often related to the research questions and inclusion criteria that did not include the components of PICO, the availability of the research question, inclusion criteria, as well as the search strategy for literature finding. The overall judgment is reported in Table 2, whereas the item-by-item assessment for each included meta-analysis is detailed in Supplementary Table S5.

### 3.3. Strength of Evidence

**Figure 2:**
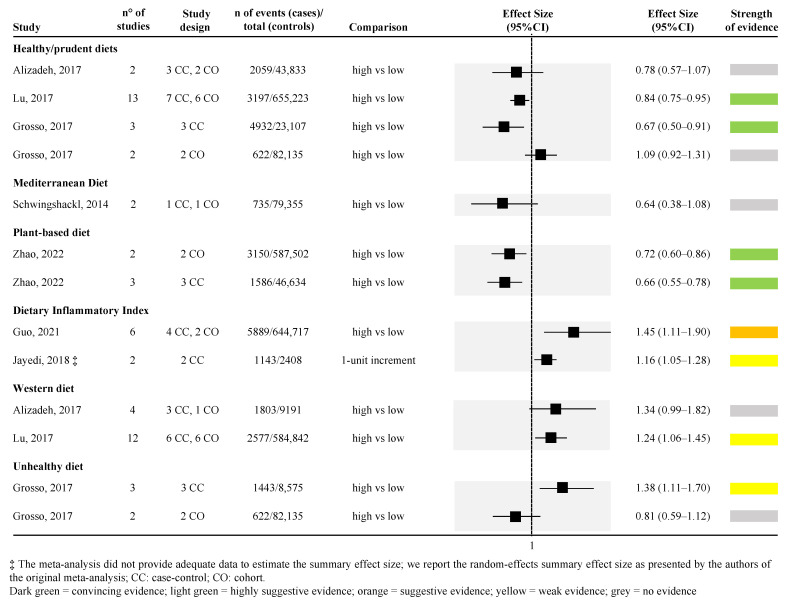
Forest plot of the meta-analyses assessing the association between dietary patterns and risk of pancreatic cancer [35,39,40,43,48,52,56].

**Figure 3:**
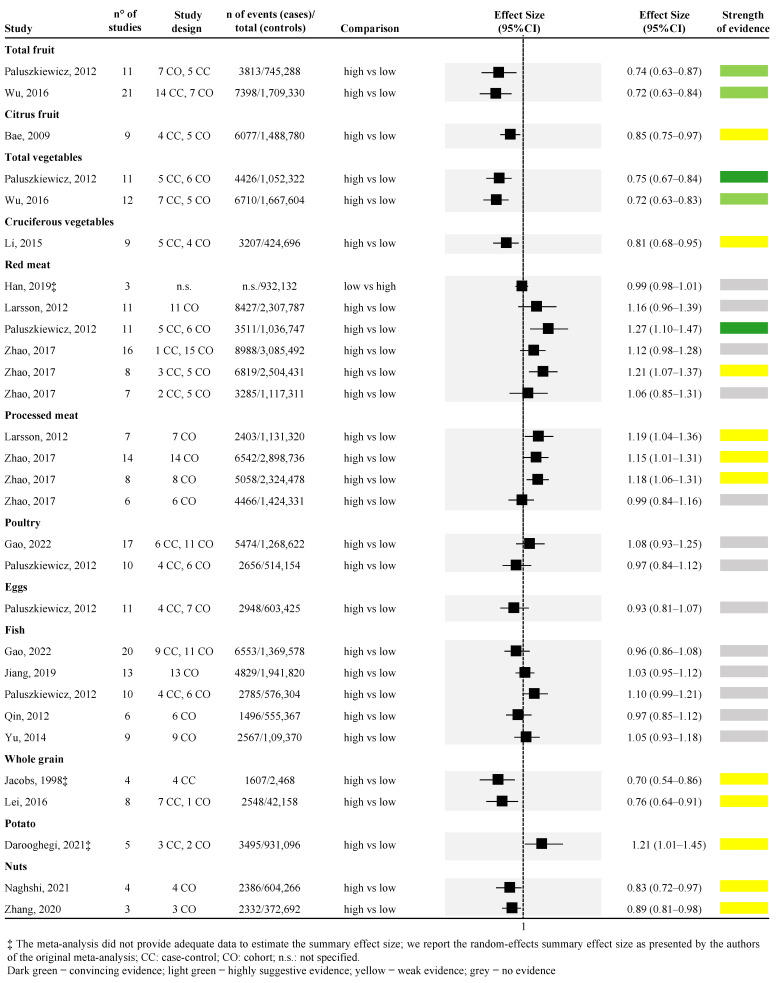
Forest plot of the meta-analyses assessing the association between dietary components and risk of pancreatic cancer [36,37,38,41,42,44,45,46,47,49,50,51,53,54,55,57].

Figure 2 and Figure 3 show the strength of evidence for each meta-analysis for dietary patterns and dietary compounds, respectively. Among all dietary patterns evaluated, none reported convincing strength of evidence. Only the association between higher adherence to healthy/prudent diets and to plant-based diets and lower PC risk reported a highly suggestive strength of evidence. However, looking specifically at the healthy/prudent diets, only two out of four meta-analyses reached this level of evidence (Figure 2) [39,48]. The remaining two did not find any level of evidence [35,39]. Among the two meta-analyses that found a highly suggestive strength of evidence, one included 13 observational studies (both case-control and cohort studies) with the largest sample size (655,223 subjects included) [random effect size: 0.84 (95% CI; 0.75; 0.95)] [48], whereas the other only included 3 case-control studies [39] (Figure 2). The relationship between plant-based diet and risk of PC was assessed in one article [56], for a total of two unique meta-analyses (the article separately reported the effect size for cross-sectional [random effect size: 0.72 (95% CI; 0.60; 0.86)] and prospective cohort studies evidence [random effect size: 0.66 (95% CI; 0.55; 0.78)]) (Figure 2). 

Higher DII (indicating a higher adherence to an inflammatory diet) was associated with a higher risk of PC in one meta-analysis, including six studies (four case-control and two cohort studies) with a suggestive strength of evidence [random effect size: 1.45 (95% CI; 1.11; 1.90)] [40]. However, another meta-analysis assessing the association between 1-unit increment in the DII and PC risk confirmed the increased risk but with a weak strength of evidence [43] (Figure 2). 

Weak strength of evidence or no evidence was found when considering western, unhealthy, and Mediterranean diets. However, in the last-mentioned case, only one meta-analysis [52], with two pooled studies, and a very small number of cases included, was retrieved (Figure 2). 

Considering the dietary components, higher consumption of vegetables was associated with a lower risk of PC; similarly, higher consumption of red meat was associated with a higher risk of PC with the highest strength of evidence. Looking at vegetable consumption, a total of two meta-analyses were retrieved [50,53] (Figure 3). One meta-analysis of eleven studies (both case-control and cohort studies) found convincing strength of evidence [random effect size: 0.75 (95% CI; 0.67; 0.84)] [50], while the other meta-analysis of twelve studies (both case-control and cohort studies) found highly suggestive strength of evidence [random effect size: 0.72 (95% CI; 0.63; 0.83)] [53] (Figure 3). Regarding red meat consumption, a total of four studies were retrieved [41,45,50,53,57] for a total of six unique meta-analyses (one article reported the effect size for men and women combined and separately for the two sexes [57]). Among the six meta-analyses, one compared the lowest versus the highest intake [41], whereas all the rest assessed the association between the highest versus the lowest intake. One meta-analysis [50], which included 11 studies, found convincing strength of evidence for the association between higher red meat consumption and higher risk of PC [random effect size: 1.27 (95% CI; 1.10; 1.47)], whereas one meta-analysis assessing the risk only among men found weak strength of evidence [random effect size: 1.21 (95% CI; 1.07; 1.37)] [57], the remaining four meta-analyses found no evidence (Figure 3).

Highly suggestive evidence was identified in two meta-analyses assessing the association between higher consumption of fruit and lower risk of PC [50,53]. The two meta-analyses included 11 and 21 observational studies, both obtaining approximately a 30% risk reduction [random effect size: 0.74 (95% CI; 0.63; 0.87), and 0.72 (95% CI; 0.63; 0.84), respectively] (Figure 3). 

The association between higher consumption of citrus fruit and lower risk of PC was assessed by one meta-analysis with a weak strength of evidence [random effect size: 0.85 (95% CI; 0.75; 0.97)] [36] (Figure 3). Similarly, one meta-analysis explored the association between higher consumption of cruciferous vegetables and lower risk of PC with a weak strength of evidence [random effect size: 0.81 (95% CI; 0.68; 0.95)] [47] (Figure 3). The association between higher intake of whole grain and lower risk of PC was assessed in two meta-analyses [42,46], both with weak strength of evidence [random effect size: 0.76 (95% CI; 0.64; 0.91)] (Figure 3). Two meta-analyses, both of which included only prospective cohort studies, with weak strength of evidence, were also found for the association between higher intake of nuts and lower risk of PC [random effect size: 0.83 (95% CI; 0.72; 0.97)] [49,55] (Figure 3). Weak strength of evidence was also found for the association between higher consumption of potatoes and higher risk of PC that was explored by one meta-analysis of both case-control and cohort studies [37] (Figure 3). Moreover, three out of the four meta-analyses, derived from two articles [45,57], found that higher consumption of processed meat was associated with a higher risk of PC with a weak strength of evidence. On the other hand, the fourth meta-analysis, which included only women, found no evidence [57] (Figure 3). 

No evidence was detected for the association between higher consumption of poultry, eggs, fish, and risk of PC, assessed by two [38,50], one [50], and five meta-analyses [38,44,50,51,54], respectively. 

Detailed information on the assessment of the strength of evidence is reported in Supplementary Tables S6 and S7.

## 4. Discussion

To our knowledge, this is the first umbrella review evaluating the quality and strength of evidence from meta-analyses on dietary risk factors for PC in adults. After a careful selection of studies according to eligibility criteria, we found 23 different systematic reviews with meta-analyses of observational studies that displayed the effect of a healthy diet, Mediterranean diet, plant-based diet, DII, western diet, unhealthy diet, and food components (e.g., total and citrus fruit, total and cruciferous vegetables, red and processed meat, poultry, eggs, fish, whole grain, nuts, and potato). Overall, assessment through the AMSTAR-2 tool found that the quality of these meta-analyses ranged from critically low to moderate, with issues mainly related to the research question and inclusion criteria, as well as the search strategy for literature finding. 

As regards dietary patterns, this umbrella review found a higher number of meta-analyses for healthy/prudent diets, whose definitions varied greatly across the included reports. Therein, convincing evidence that health/prudent diets protect against PC was reported in the studies by Lu et al. [48] and Grosso et al. [39], which respectively included the largest sample size (amongst meta-analyses evaluating dietary patterns) and one of the strongest effect sizes for a protective effect. In this sense, it is also worth noting that in the second meta-analysis, case-control studies drove the overall epidemiological link with the decreased risk of PC, whereas association in cohort studies was weaker. People on healthy/prudent diets tend to eat more vegetables, fruits, fiber, and other compounds with lower risk for PC in epidemiological studies [10]; in this sense, case-control studies are more likely to have longer follow-up periods (compared to cohort studies) in which the effect of exposure to healthy dietary components may be increased [10], and this is likely to explain the higher robustness found in our analysis. More in general, design-attributable differences in the epidemiologic study of cancer etiology have been deeply investigated, and case-control and cohort studies have complementary roles in cancer epidemiology, also considering their advantages and limitations [58]. With regards to the food-attributable risk of PC, previous evidence saw the strongest links in case-control studies rather than cohort studies, which is owing to the long latent period for cancer to develop. However, in some circumstances, cohort studies may also make a useful contribution to the evidence [10].

Grosso et al. also speculated that the geographical differences across the original studies might account for the amount and proportion of individual unhealthy food consumption, which may have affected the results of their meta-analysis [30]. It is worth also mentioning that the higher pancreatic cancer risk found in regions where dietary habits are mainly characterized by poor eating behaviors and diet quality, as well as high body mass index—such as the US, southern America, and central-eastern Europe—the hypothesis that unhealthy dietary choices may have an effect on cancer risk [17,39]. However, the literature so far available is unanimous in setting the need for further evaluation of potential geographical patterns of diets and PC [39].

When the number of individuals studied in the meta-analysis increased (particularly those enrolled in primary cohort studies), the difference according to the design was attenuated, as seen with regard to the risk according to the adherence to plant-based diets in the meta-analysis by Zhao (2022) et al. [56], which reported convincing evidence for all designs. In this sense, it is crucial to understand the role of dietary factors within the cancer process to determine the right time period for dietary assessment in cohort studies [59]. Nevertheless, possible explanations of the positive effect of plant-based diets on PC risk can be attributed to some effects of the bioactive compounds of plain foods. Indeed, these are primary sources of fiber, carotenoids, vitamins, minerals, and others that have been associated with anti-cancer properties [10,60,61].

In this respect, several studies have found possible mechanisms through which bioactive compounds could affect cancer risk. These include epigenetic mechanisms (such as downregulation of oncogenes and upregulation of tumor-suppressor genes and regulation of telomerase activity), immunoregulation, suppression of cancer cells proliferation, migration and invasion, and antioxidant and anti-inflammatory activity [60,62,63,64,65,66].

As for DII, it is well known that the pro-inflammatory potential of the diet can be a causative factor in cancer development and progression [67,68], and there was consistent and significant evidence that inflammatory status has emerged as a key mediator in PC [69]. Suggestive and weak evidence of increased risk of pancreatic cancer proportionally with higher DII score was respectively reported in the studies by Guo et al. [40] and Jayedi et al. [43], the latter enrolling less than 2500 subjects.

Weak or no evidence of an increased risk of PC was reported for western and unhealthy diets, with two studies for each analysis. Both dietary patterns are high in red and processed meat, high-fat and high-sugar dairy products, and low intakes of fruits, vegetables, and whole grains [70], which correlates to a detrimental effect on the risk of all and PCs [71,72]. 

As known, MedDiet may have a role in cancer prevention, although, in our umbrella review, it displayed no evidence for PC. Worth noting is that the role of MedDiet was evaluated only in one retrieved study [52] on the overall cancer risk in relation to adherence to MedDiet. In this meta-analysis, only two studies specifically focused on PC risk, respectively, incidence and mortality [73,74]. The small number of studies provided null results, which prevents us from drawing firm conclusions on the effect of MedDiet on PC risks, particularly if we consider that mortality outcomes are mainly affected by the treatment approaches that vary over time [75].

When exploring the association of group or single dietary components, the literature search found meta-analyses with very large populations, up to around three million people included therein. With regard to fruit consumption, the analyses found highly suggestive evidence for diets with higher total fruit consumption (two meta-analyses) and weak evidence for citrus fruit. Similarly to fruit, diets rich in vegetables showed highly suggestive-to-convincing evidence (total vegetables, two meta-analyses) or weak evidence (cruciferous vegetables, one meta-analysis). As briefly said before, fruits and vegetables are rich in vitamins, carotenoids, and other phytochemicals that have been associated with reduced risk of PC, also through a protective effect on type 2 diabetes mellitus, a well-recognized risk factor for PC [53,76].

Several overlapping biological mechanisms have been extensively suggested to shed light on the inverse relationship between the intake of whole grain foods and PC risk [10,77]: the results from our evidence assessment found only two meta-analyses, which reached a weak score, though. This should be dependably ascribed to the very low methodological quality of these two studies, assessed through AMSTAR-2 criteria. 

Meta-analyses investigating red and processed meat consumption and the risk of PC showed great variability of evidence strength and quality. Convincing evidence was reported in the study by Paluszkiewicz et al., which included quantitative analyses of PC risk in relation to main dietary compounds [50]. Conversely, the other studies reported no evidence for our assessment. Lastly, the meta-analysis by Zhao (2017) et al. reported weak evidence for diets with both high red and processed meat consumption [57]. Of note, in that study, is the fact that PC risk was significantly increased according to study design, with an overall positive association with higher consumption of red and processed meat in case-control studies but not in women in cohort studies [57]. The role of red and processed meat in carcinogenesis has been researched in great depth, and the International Agency for Research on Cancer (IARC), the cancer agency of the World Health Organization, classified processed meats as a Group 1 carcinogen (known to cause cancer) and red meat as a Group 2A carcinogen (probably causing cancer) [78]. The mechanisms by which meat correlates with cancer development are many and involve heme iron heterocyclic aromatic amines and polycyclic aromatic hydrocarbons, and nitrates and nitrites, which are found in meat, used to keep processed meat fresher for longer periods or produced by cooking [79]. 

The evidence for the other dietary components analyzed (i.e., potato, poultry, eggs, and fish) were less or not at all consistent, and the supporting literature was of low methodological quality. The slight but significantly increased PC risk associated with potato consumption has been related mainly to substances formed during its preparation and cooking. For example, fried potatoes are characterized by a high content of trans fatty acids, salt, and acrylamide. While acrylamide may exert a mutagenic effect due to the capacity of glycidamide to form DNA adducts, trans fatty acids and salt intake induce chronic inflammation, which may be related to carcinogenesis [37]. Boiled potatoes, on the other hand, have a high glycemic index. Several studies suggest that long-term consumption of high-glycemic index diets can lead to chronic hyperinsulinemia [80]. Insulin is itself a mitogen and increases the bioactivity of insulin-like growth factors (IGF-1), which can promote cancer by inhibiting apoptosis and stimulating cell proliferation [80]. As to poultry, eggs, and fish, no evidence was reported. In this context, it is important to note that the single-food approach has been increasingly replaced by the assessment of the diet as a whole through adherence indices. Indeed, dietary factors may contribute to the development of the disease, but diet as a whole has a greater impact.

Altogether, the results from this umbrella review corroborate observational findings indicating that dietary patterns emphasizing healthy/prudent diets, vegetables, fruits, whole grains, nuts, and plant-based compounds, and limiting foods with pro-inflammatory potential, red and processed meat, are associated with lower risk of PC. However, the methodological quality of the meta-analyses included in this umbrella review appears to be mainly low or critically low, fostering further research on the effect of dietary components and patterns on the risk of PC in order to draw firmer conclusions with a view to informing evidence-based practice and decision-making [81,82]. In this sense, our analyses can be useful for learning the main weakness of the current body of evidence and improving the quality of future studies on this topic [83].

To comprehensively appreciate the results of this umbrella review, some limitations must be acknowledged. First, as an umbrella review is a systematic review of meta-analyses, it depends on the quality of the included meta-analyses and does not take into account potential omissions or overlaps of original studies. Second, the retrieved evidence showed heterogeneity in terms of populations, methodologies, duration of interventions, study quality, the definition of intervention, and control diets. In particular, regardless of the type of diet, dietary pattern, and single foods within a population, dietary habits may be influenced by sociodemographic characteristics and cultural habits, which may represent potential sources of heterogeneity within the same group of dietary habits. In this sense, this umbrella review offers room for further investigating whether sociodemographic drivers may have an extent on the association between diets and PC risk. Furthermore, it highlights the lack of clinical studies on this topic. In fact, while a substantial amount of secondary research included observational studies, no analyses of RCTs on the association between diet and the risk of PC were available. Although interesting, the pooled results of observational studies are less strong due to uncontrolled multiplicity in statistical comparisons, with differences in the final results mainly due to the framing of study questions and differences in the inclusion criteria, comparisons, populations, and statistical methods used. Based on these findings, we can state that future studies need a more consistent and uniform methodology (e.g., in the assessment of eating patterns), with a detailed account of the methods used, participant sociodemographic and clinical characteristics, and outcome data. This approach would allow more precise quantification of the association between dietary patterns and PC.

## 5. Conclusions

There is a growing body of evidence regarding the association between numerous diets/dietary patterns and several cancers; however, they have different strengths of association, even if the certainty around the direction of the association is well established. More in detail, and considering results shown in the last edition of the WCRF/AICR report, evidence on dietary intake (e.g., foods, beverages, or supplements) and pancreatic cancer risk reached a limited level of evidence. Similar data are also reported, for instance, for cancer of the gallbladder, ovary, and esophagus (adenocarcinoma). On the contrary, a strong level of evidence has been reached for certain dietary factors and both the increase (red and processed meat, alcoholic drinks) and the reduction (wholegrains, foods containing dietary fiber, dairy products) of colorectal cancer risk. Based on the above-mentioned limited certainty around the association between dietary intake and pancreatic cancer risk, we developed the current umbrella review. In this umbrella review, a systematic and comprehensive literature search synthetizes meta-analyses that assessed the effects of different popular diets, dietary patterns, and food components on the risk of PC. Plant-based diets, as well as the consumption of higher fruits and vegetables, whole grains, and nuts, have been linked to the strongest and most consistent evidence, with no meta-analyses reporting negative effects. Convincing evidence of reduced risk was also reported for healthy/prudent diets, albeit not confirmed in all the found meta-analyses. DII, western patterns, and unhealthy diets reported a tendency towards detrimental effects on PC risk but showed relevant differences in terms of quality and strength of evidence. Spare meta-analyses showed weak evidence of potential risk from red and processed meat and potato. The strength of evidence for the other diet components and patterns evaluated was not statistically significant. Overall, the results from this umbrella review depict the strengths and limitations of evidence on PC risk according to the most popular diets, highlighting the need for a robust understanding of healthy eating guidance for PC prevention. 

## Figures and Tables

**Table 1:** PICOS criteria used in the present umbrella review.

Parameter	Description
**Population**	Inclusion: adults (≥18 years) Exclusion: children/adolescents and pregnant women (post-partum depression)
**Intervention**	Inclusion: all diets or dietary patterns/dietary interventions, including single food components Exclusion: study assessing the effect of supplements
**Comparison**	No intervention or any diet or dietary patterns/dietary interventions
**Outcome**	Inclusion: pancreatic cancer risk Exclusion: other outcomes or data combined for pancreatic cancer with other gastrointestinal cancers.
**Study design**	Inclusion: systematic reviews with meta-analyses of original studies (both randomized controlled trials and observational studies) Exclusion: meta-analyses not published as peer-reviewed meta-analyses in international scientific journals (book, book chapter, thesis). No full-text papers (abstract, conference paper, letter, commentary, note), systematic review without quantitative analysis, meta-analysis not reporting comprehensive data (e.g., effect size and 95% confidence intervals)

**Table 2:** Main characteristics and quantitative synthesis of meta-analyses reporting association between dietary patterns, stratified by dietary patterns and single food components, reported in alphabetical order (of the first author).

Reference	No. of Studies/Study Design	Comparison	ES	Study Population (Age ≥ 18 y)	Quality/Risk of Bias Assessment	No. of Events	Total No.	Summary ES (95% CI)		Fixed *p* Value	Random *p* Value	PI (95%)	I^2^	Quality of Meta-Analyses °	Strength of Evidence
								Fixed Effects	Random Effects						
Healthy */prudent diet															
Alizadeh, 2017 [35]	5 (3CC, 2CO)	High vs. low	OR	M/F	NOS	2059	43,833	0.85 (0.73–0.95)	0.78 (0.57–1.07)	0.029	0.122	(0.30–2.04)	71.68	Critically low	No evidence
Grosso, 2017 [39]	3CC	High vs. low	RR	M/F	JWHOFAECC	1443	8575	0.67 (0.55–0.83)	0.67 (0.50–0.91)	0.000	0.001	(0.34–1.33)	51.73	Low	Highly suggestive
Grosso, 2017 [39]	2CO	High vs. low	RR	M/F	JWHOFAECC	622	82,135	1.09 (0.92–1.29)	1.09 (0.92–1.31)	0.300	0.322	(−0.24–0.42)	11.72	Low	Highly suggestive
Lu, 2017 [48]	13 (7CC, 6CO)	High vs. low	OR	M/F	NOS	3197	655,223	0.84 (0.78–0.90)	0.84 (0.75–0.95)	0.000	0.004	(0.61–1.17)	46.82	Critically low	No evidence
**Plant-based diet**															
Zhao, 2022 [56]	2 CO	High vs. low	RR	M/F	ROBINS-I	3150	587,502	0.82 (0.75–0.89)	0.72 (0.60–0.86)	0.000	0.000	(0.46–1.13)	54.14	Low	Highly suggestive
Zhao, 2022 [56]	3 CC	High vs. low	OR	M/F	ROBINS-I	1586	46,634	0.67 (0.60–0.76)	0.66 (0.55–0.78)	0.000	0.000	(0.40–1.07)	44.80	Low	Highly suggestive
**Dietary Inflammatory Index (DII)**															
Guo, 2021 [40]	6 (4CC, 2CO)	High vs. low	RR	M/F	NOS	5889	644,717	1.19 (1.11–1.28)	1.45 (1.11–1.90)	0.000	0.006	(0.70–3.00)	88.8	Low	Suggestive
Jayedi, 2018 [43]	2CC	1-unit increment in the DII	RR	M/F	NOS	1143	2408	N.E.	1.16 (1.05–1.28)	N.E.	n.s.	N.E.	61.6	Moderate	Weak evidence
**Western diet**															
Alizadeh, 2017 [35]	4 (3CC, 1CO)	High vs. low	OR	M/F	NOS	1803	9191	1.39 (1.11–1.73)	1.34 (0.99–1.82)	0.004	0.060	(0.66–2.92)	46.92	Critically low	No evidence
Lu, 2017 [48]	12 (6CC, 6CO)	High vs. low	OR	M/F	NOS	2577	584,842	1.24 (1.14–1.35)	1.24 (1.06–1.45)	0.000	0.008	(0.69–2.22)	69.55	Critically low	Weak evidence
Unhealthy diet *															
Grosso, 2017 [39]	3CC	High vs. low	RR	M/F	JWHOFAECC	1443	8575	1.41 (1.18–1.68)	1.38 (1.11–1.70)	0.000	0.003	(0.85–2.24)	29.12	Low	Weak evidence
Grosso, 2017 [39]	2CO	High vs. low	RR	M/F	JWHOFAECC	622	82,135	0.81 (0.59–1.12)	0.81 (0.59–1.12)	0.206	0.206	(0.41–1.61)	0.00	Low	No evidence
**Mediterranean diet (MD)**															
Schwingshackl, 2014 [52]	2 (1CC, 1CO)	High vs. low	RR	M/F	NOS	735	79,355	0.76 (0.68–0.86)	0.64 (0.38–1.08)	0.000	0.095	(0.00–195.42)	89.38	Moderate	No evidence
**Total fruit**															
Paluszkiewicz, 2012 [50]	12 (7CO, 5CC)	High vs. low	RR	M/F	QRA for HOS	3813	745,288	0.76 (0.67–0.87)	0.74 (0.63–0.87)	0.000	0.000	(0.52–1.06)	29.01	Critically low	Highly suggestive
Wu, 2016 [53]	21 (14CC, 7CO)	High vs. low	RR	M/F	NOS	7398	1,709,330	0.79 (0.73–0.86)	0.72 (0.63–0.84)	0.000	0.000	(0.43–1.23)	57.67	Low	Highly suggestive
**Citrus fruit**															
Bae, 2009 [36]	9 (4CC, 5CO)	High vs. low	RR	M/F	GRADE	6077	1,488,780	0.88 (0.79–0.97)	0.85 (0.75–0.97)	0.011	0.016	(0.65–1.11)	28.99	Low	Weak evidence
**Total vegetables**															
Paluszkiewicz, 2012 [50]	11 (5CC, 6CO)	High vs. low	RR	M/F	QRA for HOS	4426	1,052,322	0.77 (0.70–0.84)	0.75 (0.67–0.84)	0.000	0.000	(0.58–0.96)	24.95	Critically low	Convincing
Wu, 2016 [53]	17 (12CC, 5CO)	High vs. low	RR	M/F	NOS	6710	1,667,604	0.76 (0.69–0.83)	0.72 (0.63–0.83)	0.000	0.000	(0.46–1.12)	45.35	Low	Highly suggestive
**Cruciferous vegetables**															
Li, 2015 [47]	9 (5CC, 4CO)	High vs. low	OR	M/F	NOS	3207	424,696	0.83 (0.75–0.92)	0.81 (0.68–0.95)	0.000	0.010	(0.52–1.25)	53.13	Moderate	Weak evidence
**Whole grain**															
Jacobs, 1998 ‡ [42]	4CC	High vs. low	OR	M/F	none	1067	2468	n.a.	0.70 (0.54–0.86)	n.a.	n.a.	N.E.	n.a.	Critically low	Weak evidence
Lei, 2016 ‡ [46]	8 (7CC, 1CO)	High vs. low	OR	M/F	NOS	2548	42,158	n.a.	0.76 (0.64–0.91)	n.a.	0.002	N.E.	11.70	Critically low	Weak evidence
**Red meat**															
Han, 2019 ‡ [41]	3	Low vs. High	RR	M/F	CATRITRB	n.s.	932,132	n.a.	0.99 (0.98–1.01)	n.a.	n.a.	N.E.	n.a.	Moderate	N.E.
Larsson, 2012 [45]	11CO	High vs. low	RR	M/F	none	8427	2,307,787	1.10 (1.00–1.21)	1.16 (0.96–1.39)	0.051	0.117	(0.60–2.18)	67.91	Critically low	No evidence
Paluszkiewicz, 2012 [50]	11 (5CC, 6CO)	High vs. low	RR	M/F	QRA for HOS	3511	1,036,747	1.25 (1.14–1.37)	1.27 (1.10–1.47)	0.000	0.001	(0.89–1.81)	46.43	Critically low	Convincing
Zhao, 2017 [57]	16 (1CC, 15CO)	High vs. low	RR	M/F	NOS	8988	3,085,492	1.15 (1.07–1.25)	1.12 (0.98–1.28)	0.000	0.090	(0.75–1.66)	51.98	Critically low	No evidence
Zhao, 2017 [57]	8 (3CC, 5CO)	High vs. low	RR	M	NOS	6819	2,504,431	1.21 (1.08–1.35)	1.21 (1.07–1.37)	0.001	0.002	(1.05–1.39)	12.99	Critically low	Weak evidence
Zhao, 2017 [57]	7 (2CC, 5CO)	High vs. low	RR	F	NOS	3285	1,117,311	1.05 (0.89–1.23)	1.06 (0.85–1.31)	0.579	0.610	(0.64–1.75)	35.45	Critically low	No evidence
**Processed meat**															
Larsson, 2012 [45]	7CO	High vs. low	RR	M/F	none	2403	1,131,320	1.19 (1.04–1.36)	1.19 (1.04–1.36)	0.011	0.011	(1.01–1.39)	0.00	Critically low	Weak evidence
Zhao, 2017 [57]	14 CO	High vs. low	RR	M/F	NOS	6542	2,898,736	1.17 (1.08–1.28)	1.15 (1.01–1.31)	0.000	0.004	(0.82–1.62)	45.67	Critically low	Weak evidence
Zhao, 2017 [57]	8 CO	High vs. low	RR	M	NOS	5058	2,324,478	1.18 (1.06–1.31)	1.18 (1.06–1.31)	0.003	0.003	(1.03–1.37)	0.00	Critically low	Weak evidence
Zhao, 2017 [57]	6 CO	High vs. low	RR	F	NOS	4466	1,424,331	0.99 (0.84–1.16)	0.99 (0.84–1.16)	0.884	0.884	(0.83–1.19)	0.00	Critically low	No evidence
**Poultry**															
Gao, 2022 [38]	17 (6CC, 11CO)	High vs. low	RR	M/F	NOS	5474	1,268,622	1.10 (1.0–1.21)	1.08 (0.93–1.25)	0.06	0.334	(1.44–3.24)	45.40	Critically low	No evidence
Paluszkiewicz, 2012 [50]	10 (4CC, 6CO)	High vs. low	RR	M/F	QRA for HOS	2656	514,154	1.00 (0.92–1.09)	0.97 (0.84–1.12)	0.966	0.662	(0.68–1.39)	34.62	Critically low	No evidence
**Eggs**															
Paluszkiewicz, 2012 [50]	11 (4CC, 7CO)	High vs. low	RR	M/F	QRA for HOS	2948	603,425	0.95 (0.89–1.01)	0.93 (0.81–1.07)	0.081	0.322	(0.66–1.33)	48.59	Critically low	No evidence
**Fish**															
Gao, 2022 [38]	20 (9CC, 11CO)	High vs. low	RR	M/F	NOS	6553	1,369,578	0.94 (0.88–1.00)	0.96 (0.86–1.08)	0.061	0.480	(0.44–0.95)	54.57	Critically low	No evidence
Jiang, 2019 [44]	13CO	High vs. low	RR	M/F	NOS	4829	1,941,820	1.03 (0.95–1.12)	1.03 (0.95–1.12)	0.471	0.471	(0.95–1.12)	0.00	Low	No evidence
Paluszkiewicz, 2012 [38]	10 (4CC, 6CO)	High vs. low	RR	M/F	QRA for HOS	2785	576,304	1.10 (1.02–1.18)	1.10 (0.99–1.21)	0.008	0.070	(0.98–1.23)	22.58	Critically low	No evidence
Qin, 2012 [51]	6CO	High vs. low	HR	M/F	Based on 4 criteria defined by the authors	1496	555,367	0.97 (0.85–1.12)	0.97 (0.85–1.12)	0.692	0.692	(0.83–1.14)	0.00	Low	No evidence
Yu, 2014 [54]	9CO	High vs. low	RR	M/F	NOS	2567	1,094,370	1.05 (0.93–1.18)	1.05 (0.93–1.18)	0.464	0.464	(0.90–1.20)	0.00	Low	No evidence
**Potato**															
Darooghegi Mofrad, 2021 ‡ [37]	5 (3CC, 2CO)	High vs. low	n.s.	M/F	ROBINS-E	3495	931,096	n.a.	1.21 (1.01–1.45)	n.a.	0.008	N.E.	n.a.	Low	Weak evidence
**Nuts**															
Naghshi, 2021 [49]	4CO	High vs. low	HR	M/F	NOS	2386	604,266	0.83 (0.72–0.97)	0.83 (0.72–0.97)	0.017	0.017	(0.67–1.04)	0.00	Moderate	Weak evidence
Zhang, 2020 [55]	3CO	High vs. low	RR	M/F	NOS	2332	372,692	0.90 (0.83–0.97)	0.89 (0.81–0.98)	0.004	0.015	(0.72–1.10)	31.46	Low	Weak evidence

° The quality of each meta-analysis was assessed using the questionnaire “A MeaSurement Tool to Assess systematic Reviews 2 (AMSTAR-2)”. * The authors of these meta-analyses used an arbitrary definition of “healthy” and “unhealthy” diets. “Healthy” or “prudent” diets included mainly fruit- and vegetable-based dietary patterns, named “healthy” or “prudent,” and some “traditional” patterns. “Unhealthy” diets were a more heterogeneous group of dietary patterns and were described as “Western,” “animal,” “fat and salty,” or “refined” and included not only animal products but also salty/sweet snacks, fatty foods, and refined foods. ‡ The meta-analysis did not provide adequate data to estimate the summary effect size; we report the random-effects summary effect size as presented by the authors of the original meta-analysis. CATRITRB: Clinical Advances through Research and Information Technology risk of bias; ES: effect size; F: female; M/F: male and female; JWHOFAECC: Joint World Health Organization–Food and Agriculture Expert Consultation Criteria; M: male; n.a.: not available; N.E.: not estimable because the authors of the original meta-analysis did not provide adequate data; NOS: New-Castle Ottawa Scale; n.s.: not specified; QRA for HOS: quantitative risk assessment for human observational studies; ROBINS-E: risk of bias in observational studies of exposures.

## Data Availability

All data are reported in the current manuscript and Supplementary Materials.

## Supplementary Materials

The following supporting information can be downloaded at: https://www.mdpi.com/article/10.3390/ijerph192214787/s1, Table S1: Literature search strategy used for each database considered; Table S2: Detailed exclusion motivations; Table S3: Inclusion/exclusion criteria reported by each included studies, declined according to the PICOS (Population, Inclusion/Exclusion, Control/Comparator and Study design); Table S4: Definition of diets/dietary patterns used in each included study; Table S5: Item-by-item methodological quality of included meta-analyses of included meta-analyses; Table S6: Assessment across the meta-analyses reporting association between dietary patterns or single food components and pancreatic cancer risk; Table S7: Bias assessment of meta-analyses reporting association between dietary patterns or single food components and pancreatic cancer risk.

## References

[1] Sung H., Ferlay J., Siegel R.L., Laversanne M., Soerjomataram I., Jemal A., Bray F. (2021). Global Cancer Statistics 2020: GLOBOCAN Estimates of Incidence and Mortality Worldwide for 36 Cancers in 185 Countries. CA Cancer J. Clin..

[2] Zhang L., Sanagapalli S., Stoita A. (2018). Challenges in diagnosis of pancreatic cancer. World J. Gastroenterol..

[3] Chen F., Childs E.J., Mocci E., Bracci P., Gallinger S., Li D., Neale R.E., Olson S.H., Scelo G., Bamlet W.R. (2019). Analysis of Heritability and Genetic Architecture of Pancreatic Cancer: A PanC4 Study. Cancer Epidemiol. Biomark. Prev..

[4] Pannala R., Basu A., Petersen G.M., Chari S.T. (2009). New-onset diabetes: A potential clue to the early diagnosis of pancreatic cancer. Lancet Oncol..

[5] Sharma A., Smyrk T.C., Levy M.J., Topazian M.A., Chari S.T. (2018). Fasting Blood Glucose Levels Provide Estimate of Duration and Progression of Pancreatic Cancer Before Diagnosis. Gastroenterology.

[6] Kirkegard J., Mortensen F.V., Cronin-Fenton D. (2017). Chronic Pancreatitis and Pancreatic Cancer Risk: A Systematic Review and Meta-analysis. Am. J. Gastroenterol..

[7] Momi N., Kaur S., Ponnusamy M.P., Kumar S., Wittel U.A., Batra S.K. (2012). Interplay between smoking-induced genotoxicity and altered signaling in pancreatic carcinogenesis. Carcinogenesis.

[8] Bracci P.M. (2012). Obesity and pancreatic cancer: Overview of epidemiologic evidence and biologic mechanisms. Mol. Carcinog..

[9] Pothuraju R., Rachagani S., Junker W.M., Chaudhary S., Saraswathi V., Kaur S., Batra S.K. (2018). Pancreatic cancer associated with obesity and diabetes: An alternative approach for its targeting. J. Exp. Clin. Cancer Res..

[10] Nucci D., Santangelo O.E., Provenzano S., Fatigoni C., Nardi M., Ferrara P., Gianfredi V. (2021). Dietary fiber intake and risk of pancreatic cancer: Systematic review and meta-analysis of observational studies. Int. J. Environ. Res. Public Health.

[11] Zheng J., Guinter M.A., Merchant A.T., Wirth M.D., Zhang J., Stolzenberg-Solomon R.Z., Steck S.E. (2017). Dietary patterns and risk of pancreatic cancer: A systematic review. Nutr. Rev..

[12] Mao Q.Q., Lin Y.W., Chen H., Qin J., Zheng X.Y., Xu X., Xie L.P. (2017). Dietary fiber intake is inversely associated with risk of pancreatic cancer: A meta-analysis. Asia Pac. J. Clin. Nutr..

[13] Mossine V.V., Mawhinney T.P., Giovannucci E.L. (2020). Dried Fruit Intake and Cancer: A Systematic Review of Observational Studies. Adv. Nutr..

[14] Higgins J.P.T., Green S. (2013). Cochrane Handbook for Systematic Reviews of Interventions. Version 5.1.0..

[15] Page M.J., McKenzie J.E., Bossuyt P.M., Boutron I., Hoffmann T.C., Mulrow C.D., Shamseer L., Tetzlaff J.M., Akl E.A., Brennan S.E. (2021). The PRISMA 2020 statement: An updated guideline for reporting systematic reviews. BMJ.

[16] Aromataris E., Fernandez R., Godfrey C.M., Holly C., Khalil H., Tungpunkom P. (2015). Summarizing systematic reviews: Methodological development, conduct and reporting of an umbrella review approach. Int. J. Evid. Based Healthc..

[17] Huang J., Lok V., Ngai C.H., Zhang L., Yuan J., Lao X.Q., Ng K., Chong C., Zheng Z.J., Wong M.C.S. (2021). Worldwide Burden of, Risk Factors for, and Trends in Pancreatic Cancer. Gastroenterology.

[18] Shea B.J., Reeves B.C., Wells G., Thuku M., Hamel C., Moran J., Moher D., Tugwell P., Welch V., Kristjansson E. (2017). AMSTAR 2: A critical appraisal tool for systematic reviews that include randomised or non-randomised studies of healthcare interventions, or both. BMJ.

[19] Cochran W.G. (1954). The combination of estimates from different experiments. Biometrics.

[20] Cochrane Collaboration (2020). Cochrane Handbook for Systematic Reviews of Interventions, Version 6.1 (Updated September 2020).

[21] Egger M., Davey Smith G., Schneider M., Minder C. (1997). Bias in meta-analysis detected by a simple, graphical test. BMJ.

[22] Sterne J.A., Sutton A.J., Ioannidis J.P., Terrin N., Jones D.R., Lau J., Carpenter J., Rucker G., Harbord R.M., Schmid C.H. (2011). Recommendations for examining and interpreting funnel plot asymmetry in meta-analyses of randomised controlled trials. BMJ.

[23] Accardi G., Shivappa N., Di Maso M., Hébert J.R., Fratino L., Montella M., La Vecchia C., Caruso C., Serraino D., Libra M. (2019). Dietary inflammatory index and cancer risk in the elderly: A pooled-analysis of Italian case-control studies. Nutrition.

[24] Genkinger J.M., Wang M., Li R., Albanes D., Anderson K.E., Bernstein L., van den Brandt P.A., English D.R., Freudenheim J.L., Fuchs C.S. (2014). Dairy products and pancreatic cancer risk: A pooled analysis of 14 cohort studies. Ann. Oncol. Off. J. Eur. Soc. Med. Oncol..

[25] Koushik A., Spiegelman D., Albanes D., Anderson K.E., Bernstein L., Van Den Brandt P.A., Bergkvist L., English D.R., Freudenheim J.L., Fuchs C.S. (2012). Intake of fruits and vegetables and risk of pancreatic cancer in a pooled analysis of 14 Cohort studies. Am. J. Epidemiol..

[26] Michaud D.S., Skinner H.G., Wu K., Hu F., Giovannucci E., Willett W.C., Colditz G.A., Fuchs C.S. (2005). Dietary patterns and pancreatic cancer risk in men and women. J. Natl. Cancer Inst..

[27] Turati F., Rossi M., Pelucchi C., Levi F., La Vecchia C. (2015). Fruit and vegetables and cancer risk: A review of southern European studies. Br. J. Nutr..

[28] Elands R.J.J., Simons C.C.J.M., Van Dongen M., Schouten L.J., Verhage B.J., Van Den Brandt P.A., Weijenberg M.P. (2016). A systematic literature review and meta-regression analysis on early-life energy restriction and cancer risk in humans. PLoS ONE.

[29] Fabiani R., Minelli L., Rosignoli P. (2016). Apple intake and cancer risk: A systematic review and meta-analysis of observational studies. Public Health Nutr..

[30] Grosso G., Micek A., Godos J., Pajak A., Sciacca S., Galvano F., Boffetta P. (2017). Health risk factors associated with meat, fruit and vegetable consumption in cohort studies: A comprehensive meta-analysis. PLoS ONE.

[31] Psaltopoulou T., Kosti R.I., Haidopoulos D., Dimopoulos M., Panagiotakos D.B. (2011). Olive oil intake is inversely related to cancer prevalence: A systematic review and a meta-analysis of 13800 patients and 23340 controls in 19 observational studies. Lipids Health Dis..

[32] Wu L., Wang Z., Zhu J., Murad A.L., Prokop L.J., Murad M.H. (2015). Nut consumption and risk of cancer and type 2 diabetes: A systematic review and meta-analysis. Nutr. Rev..

[33] Zahedi H., Djalalinia S., Asayesh H., Mansourian M., Abdar Z.E., Gorabi A.M., Ansari H., Noroozi M., Qorbani M. (2020). A higher dietary inflammatory index score is associated with a higher risk of incidence and mortality of cancer: A comprehensive systematic review and meta-analysis. Int. J. Prev. Med..

[34] Schwingshackl L., Hoffmann G. (2015). Adherence to Mediterranean diet and risk of cancer: An updated systematic review and meta-analysis of observational studies. Cancer Med..

[35] Alizadeh S., Shab-Bidar S., Mohtavinejad N., Djafarian K. (2017). A posteriori dietary patterns and risk of pancreatic and renal cancers A systematic review and meta-analysis. Nutr. Food Sci..

[36] Bae J.M., Lee E.J., Guyatt G. (2009). Citrus fruit intake and pancreatic cancer risk: A quantitative systematic review. Pancreas.

[37] Darooghegi Mofrad M., Mozaffari H., Askari M.R., Amini M.R., Jafari A., Surkan P.J., Azadbakht L. (2021). Potato Consumption and Risk of Site-Specific Cancers in Adults: A Systematic Review and Dose-Response Meta-Analysis of Observational Studies. Adv. Nutr..

[38] Gao Y., Ma Y., Yu M., Li G., Chen Y., Li X., Chen X., Xie Y., Wang X. (2022). Poultry and Fish Intake and Pancreatic Cancer Risk: A Systematic Review and Meta-Analysis. Nutr. Cancer.

[39] Grosso G., Bella F., Godos J., Sciacca S., Del Rio D., Ray S., Galvano F., Giovannucci E.L. (2017). Possible role of diet in cancer: Systematic review and multiple meta-analyses of dietary patterns, lifestyle factors, and cancer risk. Nutr. Rev..

[40] Guo Z., Hong Y., Cheng Y. (2021). Dietary inflammatory index and pancreatic cancer risk: A systematic review and dose-response meta-analysis. Public Health Nutr..

[41] Han M.A., Zeraatkar D., Guyatt G.H., Vernooij R.W.M., El Dib R., Zhang Y., Algarni A., Leung G., Storman D., Valli C. (2019). Reduction of red and processed meat intake and cancer mortality and incidence a systematic review and meta-analysis of cohort studies. Ann. Intern. Med..

[42] Jacobs D.R., Marquart L., Slavin J., Kushi L.H. (1998). Whole-grain intake and cancer: An expanded review and meta-analysis. Nutr. Cancer.

[43] Jayedi A., Emadi A., Shab-Bidar S. (2018). Dietary Inflammatory Index and Site-Specific Cancer Risk: A Systematic Review and Dose-Response  Meta-Analysis. Adv. Nutr..

[44] Jiang W., Wang M., Jiang H.Z., Chen G.C., Hua Y.F. (2019). Meta-analysis of fish consumption and risk of pancreatic cancer in 13 prospective studies with 1.8 million participants. PLoS ONE.

[45] Larsson S.C., Wolk A. (2012). Red and processed meat consumption and risk of pancreatic cancer: Meta-analysis of prospective studies. Br. J. Cancer.

[46] Lei Q., Zheng H., Bi J., Wang X., Jiang T., Gao X., Tian F., Xu M., Wu C., Zhang L. (2016). Whole Grain Intake Reduces Pancreatic Cancer Risk: A Meta-Analysis of Observational Studies. Medicine.

[47] Li L.Y., Luo Y., Lu M.D., Xu X.W., Lin H.D., Zheng Z.Q. (2015). Cruciferous vegetable consumption and the risk of pancreatic cancer: A meta-analysis. World J. Surg. Oncol..

[48] Lu P.Y., Shu L., Shen S.S., Chen X.J., Zhang X.Y. (2017). Dietary patterns and pancreatic cancer risk: A meta-analysis. Nutrients.

[49] Naghshi S., Sadeghian M., Nasiri M., Mobarak S., Asadi M., Sadeghi O. (2021). Association of Total Nut, Tree Nut, Peanut, and Peanut Butter Consumption with Cancer Incidence and Mortality: A Comprehensive Systematic Review and Dose-Response Meta-Analysis of Observational Studies. Adv. Nutr..

[50] Paluszkiewicz P., Smolińska K., Dębińska I., Turski W.A. (2012). Main dietary compounds and pancreatic cancer risk. The quantitative analysis of case-control and cohort studies. Cancer Epidemiol..

[51] Qin B., Xun P., He K. (2012). Fish or long-chain (n-3) PUFA intake is not associated with pancreatic cancer risk in a meta-analysis and systematic review. J. Nutr..

[52] Schwingshackl L., Hoffmann G. (2014). Adherence to Mediterranean diet and risk of cancer: A systematic review and meta-analysis of observational studies. Int. J. Cancer.

[53] Wu Q.J., Wu L., Zheng L.Q., Xu X., Ji C., Gong T.T. (2016). Consumption of fruit and vegetables reduces risk of pancreatic cancer: Evidence from epidemiological studies. Eur. J. Cancer Prev. Off. J. Eur. Cancer Prev. Organ. (ECP).

[54] Yu X.F., Zou J., Dong J. (2014). Fish consumption and risk of gastrointestinal cancers: A meta-analysis of cohort studies. World J. Gastroenterol..

[55] Zhang D., Dai C., Zhou L., Li Y., Liu K., Deng Y.J., Li N., Zheng Y., Hao Q., Yang S. (2020). Meta-analysis of the association between nut consumption and the risks of cancer incidence and cancer-specific mortality. Aging.

[56] Zhao Y., Zhan J., Wang Y., Wang D. (2022). The Relationship Between Plant-Based Diet and Risk of Digestive System Cancers: A Meta-Analysis Based on 3,059,009 Subjects. Front. Public Health.

[57] Zhao Z., Yin Z., Pu Z., Zhao Q. (2017). Association Between Consumption of Red and Processed Meat and Pancreatic Cancer Risk: A Systematic Review and Meta-analysis. Clin. Gastroenterol. Hepatol..

[58] Singh H., Mahmud S.M. (2009). Different study designs in the epidemiology of cancer: Case-control vs. cohort studies. Methods Mol. Biol..

[59] Flegal K.M. (1999). Evaluating epidemiologic evidence of the effects of food and nutrient exposures. Am. J. Clin. Nutr..

[60] Molina-Montes E., Salamanca-Fernandez E., Garcia-Villanova B., Sanchez M.J. (2020). The Impact of Plant-Based Dietary Patterns on Cancer-Related Outcomes: A Rapid Review and Meta-Analysis. Nutrients.

[61] Hever J., Cronise R.J. (2017). Plant-based nutrition for healthcare professionals: Implementing diet as a primary modality in the prevention and treatment of chronic disease. J. Geriatr. Cardiol..

[62] Chan J.M., Wang F., Holly E.A. (2005). Vegetable and fruit intake and pancreatic cancer in a population-based case-control study in the San Francisco bay area. Cancer Epidemiol. Biomark. Prev..

[63] Chikara S., Nagaprashantha L.D., Singhal J., Horne D., Awasthi S., Singhal S.S. (2018). Oxidative stress and dietary phytochemicals: Role in cancer chemoprevention and treatment. Cancer Lett..

[64] Gianfredi V., Vannini S., Moretti M., Villarini M., Bragazzi N.L., Izzotti A., Nucci D. (2017). Sulforaphane and Epigallocatechin Gallate Restore Estrogen Receptor Expression by Modulating Epigenetic Events in the Breast Cancer Cell Line MDA-MB-231: A Systematic Review and Meta-Analysis. J. Nutr. Nutr..

[65] Stefanska B., Karlic H., Varga F., Fabianowska-Majewska K., Haslberger A. (2012). Epigenetic mechanisms in anti-cancer actions of bioactive food components--the implications in cancer prevention. Br. J. Pharmacol..

[66] Subramaniam S., Selvaduray K.R., Radhakrishnan A.K. (2019). Bioactive Compounds: Natural Defense Against Cancer?. Biomolecules.

[67] Fowler M.E., Akinyemiju T.F. (2017). Meta-analysis of the association between dietary inflammatory index (DII) and cancer outcomes. Int. J. Cancer.

[68] Marx W., Veronese N., Kelly J.T., Smith L., Hockey M., Collins S., Trakman G.L., Hoare E., Teasdale S.B., Wade A. (2021). The Dietary Inflammatory Index and Human Health: An Umbrella Review of Meta-Analyses of Observational Studies. Adv. Nutr..

[69] Shadhu K., Xi C. (2019). Inflammation and pancreatic cancer: An updated review. Saudi J. Gastroenterol..

[70] Halton T.L., Willett W.C., Liu S., Manson J.E., Stampfer M.J., Hu F.B. (2006). Potato and french fry consumption and risk of type 2 diabetes in women. Am. J. Clin. Nutr..

[71] Mehta R.S., Song M., Nishihara R., Drew D.A., Wu K., Qian Z.R., Fung T.T., Hamada T., Masugi Y., da Silva A. (2017). Dietary Patterns and Risk of Colorectal Cancer: Analysis by Tumor Location and Molecular Subtypes. Gastroenterology.

[72] Xiao Y., Xia J., Li L., Ke Y., Cheng J., Xie Y., Chu W., Cheung P., Kim J.H., Colditz G.A. (2019). Associations between dietary patterns and the risk of breast cancer: A systematic review and meta-analysis of observational studies. Breast Cancer Res..

[73] Bosetti C., Turati F., Dal Pont A., Ferraroni M., Polesel J., Negri E., Serraino D., Talamini R., La Vecchia C., Zeegers M.P. (2013). The role of Mediterranean diet on the risk of pancreatic cancer. Br. J. Cancer.

[74] Tognon G., Nilsson L.M., Lissner L., Johansson I., Hallmans G., Lindahl B., Winkvist A. (2012). The Mediterranean diet score and mortality are inversely associated in adults living in the subarctic region. J. Nutr..

[75] Dinu M., Pagliai G., Casini A., Sofi F. (2018). Mediterranean diet and multiple health outcomes: An umbrella review of meta-analyses of observational studies and randomised trials. Eur. J. Clin. Nutr..

[76] Carter P., Gray L.J., Troughton J., Khunti K., Davies M.J. (2010). Fruit and vegetable intake and incidence of type 2 diabetes mellitus: Systematic review and meta-analysis. BMJ.

[77] Chatenoud L., Tavani A., La Vecchia C., Jacobs D.R., Negri E., Levi F., Franceschi S. (1998). Whole grain food intake and cancer risk. Int. J. Cancer.

[78] International Agency Research on Cancer (IARC) (2018). Red Meat and Processed Meat. IARC Monographs on the Evaluation of Carcinogenic Risks to Humans.

[79] Knuppel A., Papier K., Fensom G.K., Appleby P.N., Schmidt J.A., Tong T.Y.N., Travis R.C., Key T.J., Perez-Cornago A. (2020). Meat intake and cancer risk: Prospective analyses in UK Biobank. Int. J. Epidemiol..

[80] Sieri S., Krogh V. (2017). Dietary glycemic index, glycemic load and cancer: An overview of the literature. Nutr. Metab. Cardiovasc. Dis..

[81] Satija A., Bhupathiraju S.N., Spiegelman D., Chiuve S.E., Manson J.E., Willett W., Rexrode K.M., Rimm E.B., Hu F.B. (2017). Healthful and Unhealthful Plant-Based Diets and the Risk of Coronary Heart Disease in U.S. Adults. J. Am. Coll. Cardiol..

[82] Dinu M., Pagliai G., Angelino D., Rosi A., Dall’Asta M., Bresciani L., Ferraris C., Guglielmetti M., Godos J., Del Bo C. (2020). Effects of Popular Diets on Anthropometric and Cardiometabolic Parameters: An Umbrella Review of Meta-Analyses of Randomized Controlled Trials. Adv. Nutr..

[83] Gianfredi V., Nucci D., Amerio A., Signorelli C., Odone A., Dinu M. (2022). What Can We Expect from an Umbrella Review?. Adv. Nutr..

