# Supplementary material for: Diets, Dietary Patterns, Single Foods and Pancreatic Cancer Risk: An Umbrella Review of Meta-Analyses
Source: Int J Environ Res Public Health. 2022 Nov 10;19(22):14787. doi: 10.3390/ijerph192214787 (PMC9691178; doi:10.3390/ijerph192214787)
Supplement: Supplementary file 1 [file ijerph-19-14787-s001.zip › ijerph-1978841-supplementary.pdf]

Supplementary Table S1. Literature search strategy used for each database considered

Database	Syntax
PubMed/MEDLINE	"Pancreatic Neoplasms"[MeSH Terms] OR ("Pancreas"[Title/Abstract] OR "pancreatic"[Title/Abstract]) AND ("Cancer"[Title/Abstract] OR "tumor"[Title/Abstract] OR "neoplasm"[Title/Abstract] OR "cancers"[Title/Abstract] OR "tumors"[Title/Abstract] OR "neoplasms"[Title/Abstract]) AND "Diet, Food, and Nutrition"[Mesh] OR "Food"[Mesh] OR "Diet"[Mesh] OR diet* AND ((meta-analys*[tw] OR meta-analys*[tw]) OR (systematic[tw] and review*[tw]))
Scopus	(TITLE-ABS-KEY (pancreas OR pancreatic)) AND (TITLE-ABS-KEY (cancer OR neoplasm)) AND (TITLE-ABS-KEY (diet OR food OR nutrition)) AND (TITLE-ABS-KEY (meta-analys* OR meta-analys* OR systematic AND review*))
EMBASE	'pancreas tumor'/exp OR 'pancreas tumor' OR 'pancreas cancer'/exp OR 'pancreas cancer' AND 'dietary intake'/exp OR 'dietary intake' OR 'diet'/exp OR diet OR 'nutrition'/exp OR nutrition OR 'food'/exp OR food AND 'meta analysis (topic)'/exp OR 'meta analysis (topic)' OR 'systematic review (topic)'/exp OR 'systematic review (topic)'
Web of Science	(TS=(pancreatic OR pancreas)) AND TS=(cancer OR tumor OR neoplastic) AND TS=(diet OR food OR nutrition) AND TS=(meta-analys* OR meta analys* OR systematic review)
Cochrane	pancreas OR pancreatic in Title Abstract Keyword AND "Cancer" OR neoplasm OR tumor in Title Abstract Keyword AND "dietary" OR diet OR food OR nutrition in Title Abstract Keyword - (Word variations have been searched)

Supplementary Table S2. Detailed exclusion motivations

References	n	Reasons of exclusion
Genkinger, 2014; Koushik, 2012; Michaud, 2005; Turati, 2015	4	pooled analyses derived from consortium study and not systematic reviews with meta-analysis
Elands, 2016; Fabiani, 2016; Grosso, 2017; Psaltopoulou, 2011; Wu 2015; Zahedi, 2020	6	Pancreatic cancer risk not assessed separately but in combination with other gastrointestinal cancers.
Schwingshackl, 2015	1	Duplication data.

Supplementary Table S3. Inclusion/Exclusion criteria reported by each included studies, declined according to the PICOS (Population, Inclusion/Exclusion, Control/Comparator and Study design).

Author, year	Population	Intervention/exposure	Control/comparators	Outcome	Study design
Alizadeh, 2017	Inclusion: Adult (≥ 18 years)	Inclusion: Dietary patterns (Healthy and Western) assessed by FA or PCA	Inclusion: High vs. low	Inclusion: Pancreatic cancer risk	Inclusion: prospective cohorts and case-control studies
	Exclusion: n.s.	Exclusion: a priori method or statistical methods other than FA or PCA, or focused on single foods or nutrients	Exclusion: n.s.	Exclusion: n.s.	Exclusion: methodological studies, reviews, editorials, comments, cross-sectional or animal studies
Bae, 2009	Inclusion: Adult (≥ 18 years)	Inclusion: Citrus fruit	Inclusion: High vs. low	Inclusion: Pancreatic cancer risk	Inclusion: comparative epidemiological studies
	Exclusion: n.s.	Exclusion: studies not measuring the intake of citrus fruit or citrus juice at the individual level	Exclusion: n.s.	Exclusion: studies not reporting the ES of the associated measure of association	Exclusion: no original data, that is, reviews, meta-analysis;
Darooghegi Mofrad, 2021	Inclusion: Adult (≥ 18 years)	Inclusion: potato consumption, boiled, baked, roasted, mashed, or fried (potato chips or French fries)	Inclusion: High vs. low	Inclusion: Pancreatic cancer risk reported as HR, RR, or OR with 95% CI	Inclusion: cohort or case-control or pooled study designs
	Exclusion: pregnant women or children	Exclusion: potato consumption along with other food items or potatoes other than white or yellow potatoes	Exclusion: n.s.	Exclusion: Risk of cancer types other than pancreatic cancer	Exclusion: unpublished data or gray literature, such as conference articles, editorials, theses, and patents; animal, ecologic, cross-sectional studies, or RCT
Gao, 2022	Inclusion: Adult (≥ 18 years)	Inclusion: Poultry; fish	Inclusion: High vs. low	Inclusion: Pancreatic cancer risk	Inclusion: case-control or cohort studies
	Exclusion: n.s.	Exclusion: n.s.	Exclusion: n.s.	Exclusion: n.s.	Exclusion: abstracts but no full text or with no data on poultry or fish consumption
Grosso, 2017	Inclusion: Adult (≥ 18 years)	Inclusion: Adherence to a posteriori–derived dietary pattern (Healthy/prudent or Unhealthy diets)	Inclusion: High vs. low	Inclusion: Pancreatic cancer risk	Inclusion: Cohort and case–control studies
	Exclusion: n.s	Exclusion: n.s.	Exclusion: n.s.	Exclusion: Risk of cancer types other than pancreatic cancer or lack of an assessed	Exclusion: studies with lack of statistics or results

				composite outcome (eg, overall cancer mortality)	
Guo, 2021	Inclusion: Adult (≥ 18 years)	Inclusion: DII score category as exposure	Inclusion: High vs. low	Inclusion: Pancreatic cancer risk	Inclusion: RCTs, observational studies or case–control
	Exclusion: n.s.	Exclusion: DII score measured as a continuous variable	Exclusion: n.s.	Exclusion: studies without reporting multi-covariate adjusted risk estimates	Exclusion: n.s.
Han, 2019	Inclusion: Adult (≥ 18 years)	Inclusion: Red or processed meat	Inclusion: Low vs. high	Inclusion: Pancreatic cancer risk	Inclusion: Systematic reviews with meta-analyses of observational studies
	Exclusion: pregnant or subjects with a major chronic illness at baseline, or when results were not reported separately compared to those without	Exclusion: if only specific type of red meat (such as beef or lamb) or a specific type of processed meat (such as hot dogs) were assessed	Exclusion: diet intake assessed at a previous point in life (for example, if adults recalled their diet during adolescence and childhood)	Exclusion: Risk of cancer types other than pancreatic cancer; overall cancer mortality	Exclusion: n.s.
Jacobs, 1998	Inclusion: Adult (≥ 18 years)	Inclusion: Whole grain	Inclusion: High vs. low	Inclusion: Pancreatic cancer risk	Inclusion: n.s.
	Exclusion: n.s.	Exclusion: n.s.	Exclusion: n.s.	Exclusion: Risk of cancer types other than pancreatic cancer	Exclusion: n.s.
Jayedi, 2018	Inclusion: Adult (≥ 18 years)	Inclusion: the newly developed DII expressed in categories or as a continuous score	Inclusion: 1-unit increment in the Dietary Inflammatory Index	Inclusion: Pancreatic cancer risk	Inclusion: cohort or case-control studies
	Exclusion: patients with specific diseases	Exclusion: n.s.	Exclusion: n.s.	Exclusion: Risk of cancer types other than pancreatic cancer	Exclusion: cross-sectionalmstudies
Jiang, 2019	Inclusion: Adult (≥ 18 years)	Inclusion: Fish	Inclusion: High vs. low	Inclusion: Pancreatic cancer risk	Inclusion: prospective studies
	Exclusion: n.s.	Exclusion: n.s.	Exclusion: n.s.	Exclusion: risk estimates not reported HR, RR, or OR with 95% CI	Exclusion: n.s.
Larsson, 2012	Inclusion: Adult (≥ 18 years)	Inclusion: Red and/or processed meat	Inclusion: High vs. low	Inclusion: Pancreatic cancer risk	Inclusion: prospective studies
	Exclusion: n.s.	Exclusion: n.s.	Exclusion: n.s.	Exclusion: n.s.	Exclusion: n.s.

Lei, 2016	Inclusion: Adult (≥ 18 years)	Inclusion: whole grain or whole wheat foods	Inclusion: High vs. low	Inclusion: Pancreatic cancer risk	Inclusion: case-control or cohort study
	Exclusion: n.s.	Exclusion: n.s.	Exclusion: n.s.	Exclusion: risk estimates not reported as HR, RR, or OR with 95% CI	Exclusion: n.s.
Li, 2015	Inclusion: Adult (≥ 18 years)	Inclusion: Healthy/prudent diets; cruciferous vegetables	Inclusion: High vs. low	Inclusion: Pancreatic cancer risk	Inclusion: case–control or cohort study design
	Exclusion: n.s.	Exclusion: n.s.	Exclusion: n.s.	Exclusion: n.s.	Exclusion: n.s.
Lu, 2017	Inclusion: Adult (≥ 18 years)	Inclusion: Factor analysis and/or principal component analysis to identify the Western Diet or Healthy pattern	Inclusion: High vs. low	Inclusion: Pancreatic cancer risk	Inclusion: original report
	Exclusion: n.s.	Exclusion: n.s.	Exclusion: n.s.	Exclusion: n.s.	Exclusion: NA
Naghshi, 2021	Inclusion: Adult (≥ 18 years)	Inclusion: Nuts considered as tree nuts and peanuts, walnuts, pistachios, macadamia nuts, pecans, cashews, almonds, hazelnuts, and Brazil nuts, and peanut butter	Inclusion: High vs. low	Inclusion: Pancreatic cancer risk	Inclusion: observational studies with prospective, case-control, or cross-sectional designs
	Exclusion: n.s.	Exclusion: dietary pattern containing a high amount of nuts	Exclusion: n.s.	Exclusion: Risk of cancer types other than pancreatic cancer, risk of cancer recurrence, or risk estimates not reported as HR, RR, or OR with 95% CI	Exclusion: letters, comments, short communications, reviews, meta-analyses, ecological studies, and animal studies
Paluszkiewicz, 2012	Inclusion: Adult (≥ 18 years)	Inclusion: Fruits (total); vegetables (total); red meat; poultry; eggs; fish	Inclusion: High vs. low	Inclusion: Pancreatic cancer risk	Inclusion: observational studies
	Exclusion: n.s.	Exclusion: n.s.	Exclusion: n.s.	Exclusion: n.s.	Exclusion: reviews, animal studies, and experimental studies
Qin, 2021	Inclusion: Adult (≥ 18 years)	Inclusion: fish or LC-PUFA intake	Inclusion: High vs. low	Inclusion: Pancreatic cancer risk	Inclusion: cohort studies or case-control studies
	Exclusion: n.s.	Exclusion: n.s.	Exclusion: n.s.	Exclusion: risk estimates not reported as HR, RR with 95% CI	Exclusion: n.s.

Schwingshackl, 2014	Inclusion: Adult (≥ 18 years)	Inclusion: Adherence to MedDiet	Inclusion: High vs. low	Inclusion: Pancreatic cancer risk	Inclusion: cohort or case–control study design
	Exclusion: n.s.	Exclusion: n.s.	Exclusion: n.s.	Exclusion: Risk of cancer types other than pancreatic cancer	Exclusion: n.s.
Wu, 2016	Inclusion: Adult (≥ 18 years)	Inclusion: fruit, vegetable, or total fruit and vegetable intakes	Inclusion: High vs. low	Inclusion: Pancreatic cancer risk	Inclusion: observational design (e.g. cohort, nested case–control, or case–control study)
	Exclusion: n.s.	Exclusion: n.s.	Exclusion: n.s.	Exclusion: risk estimates not reported as HR, RR, or OR with 95% CI or the study reported risk estimates that could not be summarized	Exclusion: reviews without original data, ecological studies, editorials, and case reports
Yu, 2014	Inclusion: Adult (≥ 18 years)	Inclusion: Fish	Inclusion: High vs. low	Inclusion: Pancreatic cancer risk	Inclusion: prospective studies
	Exclusion: n.s.	Exclusion: frequency of fish consumption not reported	Exclusion: n.s.	Exclusion: Risk of gastrointestinal cancers other than pancreatic cancer; risk estimates not reported as HR, RR with 95% CI or the study reported risk estimates that could not be summarized	Exclusion: case-control design
Zhang, 2020	Inclusion: Adult (≥ 18 years)	Inclusion: Nuts consumption	Inclusion: High vs. low	Inclusion: Pancreatic cancer risk	Inclusion: observational or clinical design
	Exclusion: n.s.	Exclusion: nut consumption assessment performed in combination with that for other food groups	Exclusion: n.s.	Exclusion: Risk of cancer types other than pancreatic cancer	Exclusion: cross-sectional studies, animal studies, systematic reviews, meta-analyses, letters, and commentaries
Zhao, 2017	Inclusion: Adult (≥ 18 years)	Inclusion: Red and processed meat	Inclusion: High vs. low	Inclusion: Pancreatic cancer risk	Inclusion: observational studies
	Exclusion: n.s.	Exclusion: white meats and total meats without distinguishing red or processed meat were excluded	Exclusion: n.s.	Exclusion: n.s.	Exclusion: narrative reviews, systematic reviews, and meta-analyses, comments, case reports, editorials
Zhao, 2022	Inclusion: Adult (≥ 18 years)	Inclusion: Plan-based diet	Inclusion: High vs. low	Inclusion: Pancreatic cancer risk	Inclusion: cohort studies or case-control studies

	Exclusion: n.s.	Exclusion: other diet patterns	Exclusion: n.s.	Exclusion: Risk of gastrointestinal cancers other than pancreatic cancer	Exclusion: Case reports or case series, editorials, narrative reviews

CI: Confidence Interval; DII: Dietary Inflammatory Index; ES: Effect Size; FA: factor analysis, HR: hazard risk; LC-PUFA: long-chain n-3 polyunsaturated fatty acids; MedDiet: Mediterranean diet; OR: odds ratio; PCA: principal component analysis; n.s.: not specified; RCT: randomized clinical trials; RR: relative risk

Supplementary Table S4. Definition of diets/dietary patterns used in each included study

	Healthy/prudent diet	Mediterranean diet	Plant-based diet	Dietary Inflammatory Index	Western diet	Unhealthy diet
Alizadeh, 2017	High consumption of fruits, fresh vegetables, legumes, fiber, fish, poultry, whole grains, low-fat dairy products and low consumption of fat dairy, processed food and red meat	n.a.	n.a.	n.a.	High consumption of red and processed meats, sweets and desserts, soft drinks, refined grains, high-fat dairy products, fast foods and lower loading of fruits, vegetables and dietary fibers	n.a.
Grosso, 2017	Fruit or vegetable-based diets and healthy or prudent diets	n.a.	n.a.	n.a.	n.a.	Western, animal-based, diet rich in fat and salty, or with high consumption of salty/sweet snacks, fatty foods, and refined foods
Guo, 2021	n.a.	n.a.	n.a.	Literature derived, population-based diet quality index	n.a.	n.a.
Jayedi, 2018	n.a.	n.a.	n.a.	Literature derived, population-based diet quality index	n.a.	n.a.
Lu, 2017	High intake of foods such as vegetables, fruits, whole grains, olive oil, fish, soy, poultry and low-fat dairy	n.a.	n.a.	n.a.	High consumption of e.g., red and/or processed meat, refined grains, sweets, high-fat dairy products, butter, potatoes and high-fat gravy, and low intakes of fruits and vegetables	n.a.
Schwingshackl, 2014	n.a.	defined through scores that estimated the conformity of the dietary pattern (including high consumption of olive	n.a.	n.a.	n.a.	n.a.

		oil, vegetables, fruits, legumes, cereals, fish and a moderate intake of red wine during meals and low consumption of ed and processed meats and dairy products)				
Zhao, 2022	n.a.	n.a.	Fruit or vegetable-based diets, vegetarian, semi-vegetarian, pesco-vegetarian, lacto-ovo-vegetarian, vegan, Mediterranean, and healthy or prudent diets	n.a.	n.a.	n.a.

n.a.: not applicable

Supplementary Table S5. Item-by-item methodological quality of included meta-analyses

Meta-analyses	AMSTAR 2																Overall confidence
	**Q1**	**Q2**	**Q3**	**Q4**	**Q5**	**Q6**	**Q7**	**Q8**	**Q9**	**Q10**	**Q11**	**Q12**	**Q13**	**Q14**	**Q15**	**Q16**	
Alizadeh, 2017	No	Partial yes	No	Yes	No	Yes	Partial yes	Partial yes	Yes	No	Yes	No	No	No	No	Yes	Critically low
Bae, 2009	Yes	Partial yes	No	Partial yes	Yes	No	Yes	Yes	Yes	No	Yes	No	No	Yes	Yes	Yes	Low
Darooghegi Mofrad, 2021	Yes	Yes	No	Partial yes	Yes	Yes	Partial yes	Yes	Yes	No	Yes	No	Yes	Yes	Yes	Yes	Low
Gao, 2022	No	No	No	Partial yes	Yes	Yes	Partial yes	Yes	Partial yes	No	Yes	No	No	Yes	Yes	Yes	Critically low
Grosso, 2017	Yes	No	No	Partial yes	No	Yes	Partial yes	Yes	Yes	No	Yes	No	No	Yes	Yes	Yes	Low
Guo, 2021	Yes	Yes	No	Partial yes	Yes	Yes	Partial yes	Yes	Partial yes	No	Yes	No	No	Yes	No	Yes	Low
Han, 2019	Yes	Yes	No	Partial yes	Yes	Yes	Partial yes	Yes	Yes	No	Yes	Yes	Yes	Yes	Yes	Yes	Moderate
Jacobs, 1998	No	No	No	No	No	No	No	Yes	No	No	*NA*	*NA*	No	No	No	Yes	Critically low
Jayedi, 2018	Yes	Partial yes	No	Partial yes	Yes	Yes	Partial yes	Partial yes	Yes	No	Yes	Yes	Yes	Yes	Yes	Yes	Moderate
Jiang, 2019	No	Partial yes	No	Partial yes	No	No	Partial yes	Yes	Partial yes	No	Yes	Yes	Yes	Yes	Yes	Yes	Low
Larsson, 2012	No	No	No	Partial yes	No	No	No	Partial yes	Partial yes	No	Yes	No	Yes	Yes	Yes	Yes	Critically low
Lei, 2016	No	Partial yes	No	Partial yes	No	Yes	Partial yes	Partial yes	Partial yes	No	Yes	No	No	Yes	No	Yes	Critically low
Li, 2015	Yes	Partial yes	No	Partial yes	No	Yes	Partial yes	Yes	Yes	No	Yes	Yes	No	Yes	Yes	Yes	Moderate
Lu, 2017	No	No	No	No	Yes	No	Partial yes	Yes	Partial yes	No	Yes	Yes	No	Yes	No	Yes	Critically low
Naghshi, 2021	Yes	Partial yes	No	Partial yes	No	Yes	Yes	Yes	Yes	No	Yes	Yes	Yes	Yes	Yes	Yes	Moderate
Paluszkiewicz, 2012	Yes	Partial yes	No	Partial yes	No	No	Partial yes	No	Partial yes	No	Yes	No	No	Yes	No	Yes	Critically low
Qin, 2021	Yes	Partial yes	No	Partial yes	Yes	Yes	Partial yes	Yes	Yes	No	Yes	Yes	Yes	Yes	No	No	Low
Schwingshackl, 2014	Yes	Yes	No	Partial yes	Yes	No	Partial yes	Yes	Yes	No	Yes	Yes	Yes	Yes	Yes	Yes	Moderate
Wu, 2016	Yes	Partial yes	No	Partial yes	Yes	Yes	Partial yes	Partial yes	Yes	No	Yes	No	No	Yes	Yes	Yes	Low
Yu, 2014	Yes	Partial yes	No	Partial yes	No	Yes	No	Partial yes	Partial yes	No	Yes	No	Yes	No	Yes	No	Low
Zhang, 2020	Yes	Partial yes	No	Partial yes	No	Yes	Partial yes	Yes	Yes	No	Yes	No	Yes	Yes	Yes	Yes	Low
Zhao, 2017	No	Partial yes	No	Partial yes	Yes	No	Partial yes	Yes	Yes	No	Yes	No	Yes	Yes	Yes	Yes	Critically low
Zhao, 2022	Yes	Yes	No	Partial yes	Yes	Yes	Partial yes	Yes	Yes	No	Yes	No	Yes	Yes	Yes	Yes	Low

Q = Item. **Q1** = Did the research questions and inclusion criteria for the review include the components of PICO?, **Q2** = Did the report of the review contain an explicit statement that the review methods were established prior to the conduct of the review and did the report justify any significant deviations from the protocol?, **Q3** = Did the review authors explain their selection of the study designs for inclusion in the review?, **Q4** = Did the review authors use a comprehensive literature search strategy?, **Q5** = Did the review authors perform study selection in duplicate?, **Q6** = Did the review authors perform data extraction in duplicate?, **Q7** = Did the review authors provide a list of excluded studies and justify the exclusions?, **Q8** = Did the review authors describe the included studies in adequate detail?, **Q9** = Did the review authors use a satisfactory technique for assessing the risk of bias [RoB] in individual studies that were included in the review?, **Q10** = Did the review authors report on the sources of funding for the studies included in the review?, **Q11** = If meta-analysis

was performed, did the review authors use appropriate methods for statistical combination of results?, **Q12** = If meta-analysis was performed, did the review authors assess the potential impact of RoB in individual studies on the results of the meta-analysis or other evidence synthesis?, **Q13** = Did the review authors account for RoB in individual studies when interpreting/discussing the results of the review?, **Q14** = Did the review authors provide a satisfactory explanation for, and discussion of, any heterogeneity observed in the results of the review?, **Q15** = If they performed quantitative synthesis, did the review authors carry out an adequate investigation of publication bias [small study bias] and discuss its likely impact on the results of the review?, **Q16** = Did the review authors report any potential sources of conflict of interest, including any funding they received for conducting the review?

Satisfactory percentages are based on the total meta-analyses for which the dimension was judged applicable. Q1, Q4, Q9, Q11, Q12 and Q15 were used to assess the overall confidence.

Supplementary Table S6. Assessment across the meta-analyses reporting association between dietary patterns or single food components and pancreatic cancer risk

Reference	Significance threshold reached (fixed effects)	Significance threshold reached (random effects)	Number of cases	Estimate of heterogeneity >50% substantial >75 considerable	95% prediction interval	Small study effects	Strength of evidence
**Healthy diet (HD)**							
Alizadeh, 2017	<0.05	>0.05	>1000	Substantial	Including the null value	No	No evidence
Lu, 2017	<0.001	<0.001	>1000	Not large	Including the null value	No	Highly suggestive
Grosso, 2017 (CC)	<0.001	<0.001	>1000	Substantial	Including the null value	No	Highly suggestive
Grosso, 2017 (CO)	>0.05	>0.05	<1000	Not large	Including the null value	No	No evidence
**Mediterranean Diet**							
Schwingshackl, 2014	<0.001	>0.05	<1000	Considerable	Including the null value	N.E.	No evidence
**Plant-based diet**							
Zhao, 2022 (CO)	<0.001	<0.001	>1000	Substantial	Including the null value	Yes	Highly suggestive
Zhao, 2022 (CC)	<0.001	<0.001	>1000	Not large	Including the null value	No	Highly suggestive
**Dietary Inflammatory Index (DII)**							
Guo, 2021	<0.001	<0.001	>1000	Considerable	Including the null value	No	Suggestive
Jayedi, 2018	n.a.	n.a.	>1000	Substantial	N.E.	N.E.	Weak evidence
**Western diet**							
Alizadeh, 2017	<0.05	>0.05	>1000	Substantial	Including the null value	No	No evidence

Lu, 2017	<0.001	<0.05	>1000	Substantial	Including the null value	No	Weak evidence
**Unhealthy diet**							
Grosso, 2017 (CC)	<0.001	<0.05	>1000	Not large	Including the null value	Yes	Weak evidence
Grosso, 2017 (CO)	>0.05	>0.05	<1000	Not large	Including the null value	Yes	No evidence
**Total fruit**							
Paluszkiewicz, 2012	<0.001	<0.001	>1000	Not large	Including the null value	No	Highly suggestive
Wu, 2016	<0.001	<0.001	>1000	Substantial	Including the null value	No	Highly suggestive
**Citrus fruit**							
Bae, 2009	<0.05	<0.05	>1000	Not large	Including the null value	Yes	Weak evidence
**Total vegetables**							
Paluszkiewicz, 2012	<0.001	<0.001	>1000	Not large	Excluding the null value	No	Convincing
Wu, 2016	<0.001	<0.001	>1000	Not large	Including the null value	No	Highly suggestive
**Cruciferous vegetables**							
Li, 2015	<0.001	<0.05	>1000	Substantial	Including the null value	No	Weak evidence
**Red meat**							
Han, 2019‡	n.a	n.a.	n.a.	n.a.	n.a.		N.E.
Larsson, 2012	>0.05	>0.05	>1000	Substantial	Including the null value	No	No evidence
Paluszkiewicz, 2012	<0.001	<0.001	>1000	Not large	Excluding the null value	No	Convincing
Zhao, 2017 (M/F)	<0.001	>0.05	>1000	Substantial	Including the null value	No	No evidence
Zhao, 2017 (M)	<0.001	<0.05	>1000	Not large	Excluding the null value	No	Weak evidence
Zhao, 2017 (F)	>0.05	>0.05	>1000	Not large	Including the null value	No	No evidence

**Processed meat**							
Larsson, 2012	<0.05	<0.05	>1000	Not large	Excluding the null value	No	Weak evidence
Zhao, 2017 (M/F)	<0.001	<0.05	>1000	Not large	Including the null value	No	Weak evidence
Zhao, 2017 (M)	<0.05	<0.05	>1000	Not large	Excluding the null value	No	Weak evidence
Zhao, 2017 (F)	>0.05	>0.05	>1000	Not large	Including the null value	No	No evidence
**Poultry**							
Gao, 2022	>0.05	>0.05	>1000	Not large	Excluding the null value	No	No evidence
Paluszkiewicz, 2012	>0.05	>0.05	>1000	Not large	Including the null value	No	No evidence
**Eggs**							
Paluszkiewicz, 2012	>0.05	>0.05	>1000	Not large	Including the null value	No	No evidence
**Fish**							
Gao, 2022	>0.05	>0.05	>1000	Not large	Excluding the null value	No	No evidence
Jiang, 2019	>0.05	>0.05	>1000	Not large	Including the null value	No	No evidence
Paluszkiewicz, 2012	<0.05	≥0.05	>1000	Not large	Including the null value	No	No evidence
Qin, 2012	>0.05	>0.05	>1000	Not large	Including the null value	No	No evidence
Yu, 2014	>0.05	>0.05	>1000	Not large	Including the null value	No	No evidence
**Whole grain**							
Jacobs, 1998‡	n.a.	n.a.	>1000	n.a.	n.a.	n.a.	Weak evidence
Lei, 2016‡	n.a.	<0.05	>1000	Not large	n.a.	n.a.	Weak evidence
**Potato**							
Darooghegi Mofrad‡	n.a.	<0.05	>1000	n.a.	n.a.		Weak evidence
**Nuts**							

Naghshi, 2021	<0.05	<0.05	>1000	Not large	Including the null value	No	Weak evidence
Zhang, 2020	<0.05	<0.05	>1000	Not large	Including the null value	No	Weak evidence

‡The meta-analysis did not provide adequate data to estimate the summary effect size; we report the random-effects summary effect size as presented by the authors of the original meta-analysis. n.a. = not available; NE = not estimable because less than 3 studies were available for each meta-analysis or because data not available

Supplementary Table S7. Bias assessment of meta-analyses reporting association between dietary patterns or single food components and pancreatic cancer risk

Reference	Largest study (95% CI)	SE	Egger test p-value
**Healthy diet (HD)**			
Alizadeh, 2017	1.02 (0.81; 1.24)	0.11	0.40
Lu, 2017	0.84 (0.72-0.98)	0.07	0.54
Grosso, 2017 (CC)	0.55 (0.35-0.86)	0.23	0.96
Grosso, 2017 (CO)	1.25 (0.84-1.86)	0.20	0.46
**Mediterranean diet**			
Schwingshackl	0.82 (0.72-0.93)	0.07	N.E.
**Plant-based diet**			
Zhao, 2022 (CO)	0.35 (0.17-0.73)	0.06	0.00
Zhao, 2022 (CC)	0.93 (0.71-1.23)	0.14	0.41
**Dietary Inflammatory Index (DII)**			
Guo, 2021	1.24 (1.11-1.38)	0.05	0.17
Jayedi, 2018	n.a.	n.a.	n.a.
**Western diet**			
Alizadeh, 2017	0.71 (0.26-1.16)	0.23	0.12
Lu, 2017	1.28 (1.04-1.57)	0.11	0.98
**Unhealthy diet**			
Grosso, 2017 (CC)	0.52 (0.14-0.88)	0.18	0.03
Grosso, 2017 (CO)	0.74 (0.48-1.14)	0.22	0.02
**Total fruit**			
Paluszkiewicz, 2012[a]	1.02 (0.61-1.09)	0.15	0.02
Wu, 2016[a]	0.89 (0.77-1.04)	0.08	0.01
**Citrus fruit**			
Bae, 2009	0.00 (-0.20; 0.20)	0.10	0.08
**Total vegetables**			
Paluszkiewicz, 2012	0.83 (0.75-1.01)	0.07	0.38
Wu, 2016[a]	1.02 (0.84-1.24)	0.10	0.09
**Cruciferous vegetables**			
Li, 2015	0.9 (0.63-0.91)	0.09	0.32
**Red meat**			
Han, 2019‡	n.a.	n.a.	n.a.

Larsson, 2012	1.11(0.92-1.33)	0.09	0.90
Paluszkiewicz, 2012	1.00 (0.94-1.38)	0.09	0.64
Zhao, 2017 (M/F)	1.22 (1.01-1.47)	0.09	0.26
Zhao, 2017 (M)	1.1 (0.9-1.34)	0.10	0.49
Zhao, 2017 (F)	1.01 (0.74-1.38)	0.16	0.54
**Processed meat**			
Larsson, 2012	1.09 (0.86-1.38)	0.07	0.53
Zhao, 2017 (M/F)	1.22 (1.01-1.47)	0.09	0.56
Zhao, 2017 (M)	1.33 (1.11-1.59)	0.09	0.85
Zhao, 2017 (F)	0.97 (0.73-1.29)	0.15	0.79
**Poultry**			
Gao, 2022	0.16 (-0.04; 0.36)	0.01	0.94
Paluszkiewicz, 2012	1.03 (0.93-1.14)	0.05	0.43
**Eggs**			
Paluszkiewicz, 2012	0.95 (0.89-1.03)	0.04	0.80
**Fish**			
Gao, 2022	0.89 (0.77-1.02)	0.07	0.49
Jiang., 2019	1.07 (0.92-1.24)	0.08	0.70
Paluszkiewicz, 2012	1.02 (0.92-1.12)	0.05	0.78
Qin, 2012	0.91 (0.75-1.11)	0.10	0.39
Yu, 2014	0.91 (0.75-1.11)	0.10	0.31
**Potato**			
Darooghegi Mofrad‡	n.a.	n.a.	n.a.
**Whole grain**			
Jacobs, 1998‡	n.a.	n.a.	n.a.
Lei, 2016‡	n.a.	n.a.	n.a.
**Nuts**			
Naghshi, 2021	1.14(0.58-2.25)	0.18	0.86
Zhang, 2020	0.93 (0.85-1.03)	0.05	0.36

‡The meta-analysis did not provide adequate data to estimate the summary effect size; we report the random-effects summary effect size as presented by the authors of the original meta-analysis. [a] The Egger test was statistically significant ($p<0.10$) but the largest study had larger effect size compared to the summary effect size under random effects, denoting the absence of small-study effects

n.a.: not available; N.E. = not estimable because less than 3 studies were available for each meta-analysis.
